# Mindfulness-based training with transcranial direct current stimulation modulates neuronal resource allocation in working memory: A randomized pilot study with a nonequivalent control group

**DOI:** 10.1016/j.heliyon.2018.e00685

**Published:** 2018-07-11

**Authors:** Michael A. Hunter, Gregory Lieberman, Brian A. Coffman, Michael C. Trumbo, Mikaela L. Armenta, Charles S.H. Robinson, Matthew A. Bezdek, Anthony J. O'Sickey, Aaron P. Jones, Victoria Romero, Seth Elkin-Frankston, Sean Gaurino, Leonard Eusebi, Eric H. Schumacher, Katie Witkiewitz, Vincent P. Clark

**Affiliations:** a Psychology Clinical Neuroscience Center, The University of New Mexico, Albuquerque, NM, USA; b Department of Psychology, The University of New Mexico, Albuquerque, NM, USA; c The Mind Research Network and LBERI, Albuquerque, NM, USA; d The Center for Brain Recovery and Repair, The University of New Mexico Health Sciences Center, NM, USA; e Department of Neurosciences, The University of New Mexico, Albuquerque, NM, USA; f U.S. Army Research Laboratory, Aberdeen Proving Ground, MD, USA; g Department of Bioengineering, The University of Pennsylvania, Philadelphia, PA, USA; h Department of Psychiatry, The University of Pittsburgh School of Medicine, Pittsburgh, PA, USA; i School of Psychology, Georgia Institute of Technology, Atlanta, GA, USA; j Charles River Analytics, Cambridge, MA, USA

**Keywords:** Psychology, Clinical psychology, Neuroscience

## Abstract

Mindfulness-based training (MBT) and transcranial electrical stimulation (TES) methods such as direct current stimulation (tDCS) have demonstrated promise for the augmentation of cognitive abilities. The current study investigated the potential compatibility of concurrent “electrical” MBT and tDCS (or eMBT) by testing its combined effects on behavioral and neurophysiological indices of working memory (WM) and attentional resource allocation. Thirty-four healthy participants were randomly assigned to either a MBT task with tDCS group (eMBT) or an active control training task with sham tDCS (Control) group. Training lasted 4-weeks, with up to twenty MBT sessions and with up to eight of those sessions that were eMBT sessions. Electroencephalography was acquired during varying WM load conditions using the n-back task (1-, 2-, 3-back), along with performance on complex WM span tasks (operation and symmetry span) and fluid intelligence measures (Ravens and Shipley) before and after training. Improved performance was observed only on the 3-back and spatial span tasks for eMBT but not the Control group. During 3-back performance in the eMBT group, an increase in P3 amplitude and theta power at electrode site Pz was also observed, along with a simultaneous decrease in frontal midline P3 amplitude and theta power compared to the Control group. These results are consistent with the neural efficiency hypothesis, where higher cognitive capacity was associated with more distributed brain activity (i.e., increase in parietal and decrease in frontal amplitudes). Future longitudinal studies are called upon to further examine the direct contributions of tDCS on MBT by assessing the differential effects of electrode montage, polarity, current strength and a direct contrast between the eMBT and MBT conditions on performance and neuroimaging outcome data. While preliminary, the current results provided evidence for the potential compatibility of using eMBT to modulate WM capacity through the allocation of attention and its neurophysiological correlates.

## 1 Introduction

While neural plasticity refers to the brain's capacity for structural and neurophysiological changes in response to environmental demands (Lövdén et al., 2010), cognitive plasticity refers to alterations in performance related to systems-level functionality (Mercado, 2008). Ultimately, functional changes, such as increased dependence on executive functioning, in turn influences neuroplasticity (Greenwood and Parasuraman, 2010). Given that neural and cognitive plasticity are interactive and dynamic processes (Thelen and Smith, 1996; Stiles, 2000), neurocognitive augmentation techniques can be used to enhance systems-level functionality and facilitate neuroplastic change by targeting domain-general cognitive networks.

Cognitive control is a primary target for neurocognitive augmentation techniques as it guides voluntary and complex behavior, which involves the efficient use of prefrontal neuronal resources in the face of high cognitive demands (see Miller and Cohen, 2001 for review). Working memory (WM) is closely related to cognitive control processes, which stores and manipulates transitory information necessary for complex tasks such as learning and reasoning (see Shah and Miyake, 1999). The central executive component of WM engages attention to assist the memory system in maintaining goal-directed information in the face of distraction to favor (or bias toward) task-relevant stimuli (Miller and Cohen, 2001; Shah and Miyake, 1999; Braver, 2012; Engle and Kane, 2004; Missonnier et al., 2006).

### 1.1 Defining and targeting the control and allocation of attention

Working memory demand is dependent upon the ability to focus attention on incoming stimuli by exerting top-down control over items that occupy a very limited space. This attentional control further depends upon the efficient allocation of neuronal resources to achieve the varying demands of a task (Owen et al., 2005; Shipstead et al., 2012). Cognitive load can be examined in a monotonically dose-related fashion by varying the number of items to be maintained in this limited space. The classical n-back task has been widely used as an assessment of WM load (Schmiedek et al., 2009), which requires increased levels of attentional control and memory processes to continuously and simultaneously evaluate and maintain goal-relevance of information (Miyake and Friedman, 2012; Barrett et al., 2004). So, by increasing the number of items to be maintained in WM (e.g., from 1- to 3-back conditions), a higher demand of resources is required, predicting that high WM performers are better able to extend that resource on a continuing basis (see McCabe et al., 2010 for review). Thus, individual differences in WM capacity are closely related to the allocation of cortical resources involved in effectively engaging the central executive attention system for successful maintenance of task goals (McCabe et al., 2010; Drew et al., 2006).

#### 1.1.1 Training the allocation of attentional demands via mindfulness meditation

During mindfulness training, practitioners cultivate a nonjudgmental state of attention to experiences in the present moment (Kabat-Zinn et al., 1985), resulting in the active engagement of attentional control abilities (Brown and Ryan, 2003; Lutz et al., 2008; Malinowski, 2013). Mindfulness-based training (MBT) has been operationalized into two distinct types of practices, focused attention (FA) and open monitoring (OM) (Hölzel et al., 2011). In FA, practitioners are trained to direct and focus attention toward an object, such as the breath or the body. To maintain the goal of the task, individuals are trained to recognize when they are off-task (e.g., mind-wandering) and then redirect their attention back to the object of attention, without judgment. For example, in a breath awareness FA task a practitioner may be instructed: “If your mind wanders 100 times, just bring your attention back to the breath 100 times, redirecting the attention back to the breath without judgment.” For the OM technique, practitioners are open to perceiving and observing any sensations or thoughts, thereby allowing a flexible, unrestricted flow of attention (Hölzel et al., 2011).

Working memory performance has been shown to improve after multiple MBT sessions (Cahn and Polich, 2009; Jha et al., 2010; Mrazek et al., 2013; Zeidan et al., 2010), making MBT a technique for targeting domain-general attentional control processes. For example, Zeidan et al. (2010) implemented a relatively short MBT protocol (4 days of training, 20 minutes each day) and compared adaptive 2-back performance before and after training. Results showed an increase in extended hit rate accuracy (i.e., the number of accurate and consecutive working memory discriminations) in the MBT group compared to an active control group. The authors concluded that the MBT group maintained focus and accurately retrieved information from WM under conditions that required rapid stimulus processing (Zeidan et al., 2010).

Similarly, previous MBT studies have reported a reduction of P3 amplitude to distractor stimuli during attention-related task performance (Slagter et al., 2007; Cahn and Polich, 2009; Tang et al., 2009; Moore et al., 2012; Cahn et al., 2013). Concordantly, increased frontal theta during rest (Tang et al., 2009) and the meditative state (Baijal and Srinivasan, 2009) have been observed after MBT, further suggesting an enhancement for efficiently allocating attention. Moreover, in line with the hypothesis that MBT engages the attentional control brain networks—the same mechanisms that the n-back task evokes, Course-Choi et al. (2017) found that pairing MBT with n-back training significantly improved performance on cognitive control tasks (including an n-back task) compared to performance after just n-back training alone, suggesting that MBT may add to processing efficiency of WM.

#### 1.1.2 Facilitating the benefits of mindfulness-based training and its neuroplastic change via non-invasive brain stimulation

Non-invasive brain stimulation has become a widespread method for modulating neuronal excitability. Combining cognitive training with brain stimulation may facilitate the mechanisms involved in neuroplasticity, which further allows for and/or results from an acceleration of learning (Clark and Parasuraman, 2014). Transcranial direct current stimulation (tDCS), a low-cost, portable, non-invasive brain stimulation technique, has been employed to modulate the excitability of functional brain networks (Nitsche and Paulus, 2000; Polanía et al., 2011; Keeser et al., 2011; Peña-Gómez et al., 2012; Hunter et al., 2013). A single-session of tDCS is thought to acutely increase the membrane potential for spontaneous firing of cortical neurons near the anode electrode (Nitsche and Paulus, 2000; Dieckhöfer et al., 2006), while decreasing membrane potential excitability near the cathode (Nitsche and Paulus, 2000; Dieckhöfer et al., 2006). This is followed by lasting effects that are NMDA-receptor-dependent (Liebetanz et al., 2002), similar to long-term potentiation (LTP) and long-term depression (LTD), which are posited to underlie learning and memory (Rioult-Pedotti et al., 1998). In relation to attention and brain modulation, tDCS has been used to improve the effectiveness of: threat detection (Clark et al., 2012), WM training (Trumbo et al., 2016), attentional bias training (Clarke et al., 2014) and the re-allocation of attentional resources under varying cognitive load conditions in an executive attention task (Weiss and Lavidor, 2012).

#### 1.1.3 Combining mindfulness-based training with non-invasive electrical stimulation

We propose that tDCS can be paired with MBT (eMBT), which together will facilitate ongoing neural patterns of activity associated with controlling and regulating attention. The right prefrontal cortex is a major (domain-general) hub of the frontal-parietal control network due to its involvement in top-down attentional control (see Corbetta and Shulman, 2002; Petersen and Posner, 2012; Hugdahl et al., 2015 for reviews). The right inferior frontal gyrus (IFG) has been shown to play a specific role in controlling attention toward goal-specific, salient cues (Hampshire et al., 2009) by serving as a top-down biasing signal for maintaining and executing goal-directed behavior (Hampshire et al., 2010). Our previous findings showed that anodal stimulation of right IFG at 2.0 mA for 30 minutes led to accelerated learning (Clark et al., 2012) and enhanced attention (Coffman et al., 2012). Moreover, regional resting-state functional connectivity changes in right IFG have been reported after 40 days of MBT (Yang et al., 2016), as well as increases in right IFG cortical thickness (Lazar et al., 2005).

However, there is an ongoing controversy surrounding the issue of selecting the optimal stimulation protocol to produce robust and reliable effects on cognitive abilities (see Horvath et al., 2014 for review). For example, previous meta-analyses have concluded that the effects of tDCS on WM are small (Hill et al., 2016), partial (Brunoni and Vanderhasselt, 2014), or nonexistent (Horvath et al., 2015). A meta-analysis made a provisional conclusion that while anodal stimulation of the left DLPFC (1–2 mA) can improve WM performance, this effect was small, suggesting a more optimal tDCS parameter space (Mancuso et al., 2016). Furthermore, there is a differential influence of current dose and brain pathology (Cf. Hoy et al., 2013, Hoy et al., 2014), as well as participant characteristics (e.g., levels of motivation) and the type of task used (easy vs. difficult), which can influence the efficacy of tDCS (see Berryhill et al., 2014 for review). To this end, the precise tDCS parameter space under which tDCS may exert the most optimal improvements has yet to be fully examined in any cognitive domain.

Recently, “E-meditation” has been proposed as a novel approach to potentially reduce the learning curve of intensive meditative sessions and to possibly simultaneously enhance psychological well-being (Badran et al., 2017) and emotional intelligence and emotional valence (Robinson et al., 2017). For example, Badran et al., 2017 conducted a cross-over double-blind pilot study, which combined 20 mins of guided meditation (audio recordings) with tDCS at 1.0 mA, 2.0 mA and a sham stimulation conditions. The outcome measures were self-reports of mindfulness and affect before and after each of the 3 (total) sessions over the course of 3 weeks. The anode electrode was placed over F8 to facilitate task-specific learning and the cathode electrode was placed over the contralateral supraorbital region with the hypothesis of “electrically” inducing a transient hypofrontality. While Badran et al. (2017) found statistically non-significant differences in mood ratings, self-endorsements of mindfulness increased in the stimulation groups compared to sham. More recently, Robinson et al. (2017) conducted a pilot study designed to enhance emotional intelligence and valence using loving kindness meditation (LKM) and two different types of tDCS protocols. One protocol placed the anode electrode over the left lateral prefrontal cortex (DLPFC; F3) and the other protocol placed the anode over the right temporoparietal junction (TPJ; CP6). For both protocols, the cathode was placed on the contralateral upper arm. Subjective reports on pre-selected images from the International Affective Picture System were used as the primary outcome measures of emotional valence (positive vs. negative). This study found an increase in positive affect in the LKM groups which received both active and sham tDCS to the right TPJ. While this study confirmed the beneficial effects of LKM compared to a control group, it was uncertain how tDCS to the right TPJ, independent of active vs. sham conditions, contributed to this effect. It is also important to note that the left DLPFC stimulation did not have any statistically significant effects on the outcome measures. Both these studies demonstrated the feasibility of tDCS with meditation and the possible utility for targeting certain domains of the meditative process.

However, the interpretation of these studies is limited by the sole use of self-reports for a single-session of eMBT. It is possible that a combination of a multi-session MBT with a tDCS montage to specifically target a domain-general attentional hub may produce measurable effects. As described in detail in the next section, the present study did not rely on self-reports, but rather collected dependent measures that are thought to be more objective and reliable indicators of the cognitive domain of interest.

#### 1.1.4 Objective outcome measures: time-domain electrophysiological correlates of attentional allocation in working memory load

The characterization of event-related potential (ERP) components using electroencephalography (EEG) have provided useful information regarding WM load and resource allocation (Polich, 2007). The P3, a positive ERP component that peaks ∼300–600 ms after a novel, or target stimulus onset, is thought to be a primary marker of context evaluation and subsequent allocation of attentional resources (see Kok, 2001 for review). More specifically, the P3 complex is thought to index the integration of information into functionally relevant brain networks that subserve attentional features of a task and related memory storage processes (Kok, 2001; Soltani and Knight, 2000). To this end, the P3 complex has been dissociated into two major physiologically and phenomenologically distinct components, the P3a and P3b and a number of subcomponents, each composed of distinct spatial topologies. The P3a has a frontocentral topography and is likely generated in prefrontal brain regions during the evaluation and categorization of incoming stimuli, such as maintaining stimulus information and processing stimulus novelty (Gevins and Smith, 2000; Soltani and Knight, 2000; Polich and Criado, 2006; Bledowski et al., 2004). The P3b has a relatively more posterior topography (depending on other task and stimulus details) and is generated in temporal-parietal areas (Bledowski et al., 2004), which are involved in memory-related storage processes.

Together, the amplitude of the P3 complex is thought to reflect task-specific activations in an event-categorization network that is controlled by the joint operation of attention and working memory (see Kok, 2001 for review). Moreover, the magnitude of the P3 complex has been consistently shown to decrease as a function of WM load, indicating a reallocation of attentional resources to accommodate increased capacity demands to distributed networks (see Kok, 2001; Polich, 2007 for reviews; Dong et al., 2015; Saliasi et al., 2013). Notably, increased posterior P3 amplitude has been proposed as a possible candidate marker of training-related transfer of processing speed and cognitive control (Oelhafen et al., 2013).

#### 1.1.5 The time-frequency electrophysiological correlates of attentional allocation in working memory load

Theta power (4–8 Hz) is thought to govern the relationship between attentional control (P3a) and memory processes (P3b) (Polich, 2007; Polich and Criado, 2006). More specifically, theta-band power has been shown to reflect the coordination and integration of frontal and parietal neuronal populations involved in the internal mental context for top-down processing (Sarnthein et al., 1998; Von Stein and Sarnthein, 2000; Sauseng et al., 2005, 2010; Reinhart et al., 2015). Thus, the examination of frontal and parietal theta power in the P3 range overlaps with the time interval of stimulus processing, integration and categorization of information into functionally relevant brain networks (Polich, 2007), such as integration into frontal-parietal cortical networks during complex WM processing (Sauseng et al., 2005, Sauseng et al., 2010, see Gevins et al., 1997 for review).

Previous studies found an increase in frontal theta power as a function of WM load during the n-back task and suggested that frontal theta could be utilized as an index of WM load (Gevins et al., 1997; see Klimesch, 1999 for review; Meltzer et al., 2008; Hsieh and Ranganath, 2014; Itthipuripat et al., 2013). P3-range theta power during the n-back is thought to represent the EEG correlate of focused attention to target stimuli (Missonnier et al., 2006). Previous studies have shown that theta power is specifically increased over frontal cortex for successful versus failed WM manipulation (Itthipuripat et al., 2013) and that posterior midline theta power is increased as a function of WM load, which is thought to serve as the integrative marker of memory trace matching between top-down and bottom-up visual information processing (Sauseng et al., 2010). Theta power has also been associated with the effects of meditation, further indicating a self-induced top-down regulation of attentional processing (see Polich, 2007 for review).

#### 1.1.6 Evaluation of near and far transfer

Lastly, within the broader context of evaluating the efficacy of cognitive training techniques, the goal of cognitive training is to improve cognitive abilities rather than simply improving performance specific to a trained task (Cormier and Hagman, 1987; Klingberg, 2010; Shipstead et al., 2012; Enriquez-Geppert et al., 2013). Transfer tasks must be employed to assess whether the cognitive construct has been improved and not just the stimulus mappings specific to a task. Complex WM span tasks, such as the operation span (O-span) and symmetry span (S-span) tasks, were initially developed to measure the executive attention component of WM and have been shown to correlate with performance on tasks that require top-down guidance of attention (Chow and Conway, 2015; Hofmann et al., 2012; Unsworth et al., 2014). More specifically, in complex WM tasks, to-be-remembered items are interspersed with distractor events, such as solving math problems or evaluating the symmetry of spatial figures. Both tasks require the engagement of attention to select and actively maintain items in the presence of internal and external distraction (Unsworth et al., 2005). These tasks have been used as near transfer tasks to assess the effects of WM training alone (Baniqued et al., 2015), WM training with tDCS (Richmond et al., 2014; Trumbo et al., 2016) and WM-related effects due to MBT (Zeidan et al., 2010). Furthermore, to assess possible effects on a broader general fluid intelligence ability, the Ravens Progressive Matrices (Raven, 2003) and Shipley IQ (Shipley et al., 2009) tests have been used in previous studies (Richmond et al., 2014). Therefore, we hypothesized that training-related behavioral gains and a re-distribution of neurophysiological responses observed in the n-back would positively correlate with performance on the complex WM and fluid IQ tasks, indicating task transfer.

### 1.2 Objectives and hypotheses

The objective of the current study was to examine the combined effects of MBT with right prefrontal tDCS by measuring varying levels of WM performance and its EEG neural correlates of resource allocation. The excellent temporal resolution provided by EEG makes this method ideal for studying the dynamic processes that underlie the attentional reallocation of neuronal resources at midline frontal (Fz), parietal (Pz) and anodal stimulation electrode (F10) sites, which can be compared in eMBT relative to baseline and an active control training group with sham stimulation (Control). We hypothesized that the eMBT group would exhibit improved WM capacity during the n-back task as reflected by higher accuracy and faster response times (RTs) compared to the Control condition, particularly at higher WM load conditions, reflecting enhanced attentional resource allocation. Given the multi-session design of this study to cultivate learning of an adaptive skill by the continual engagement of specific attentional brain networks–in contrast to a single-session design to activate a transient brain state, we also hypothesized a post-training re-distribution of frontal and parietal P3 amplitude and theta power in the eMBT group compared to baseline and the control group, indicating more efficient neural processing (Neubauer and Fink, 2009). That is, while we would hypothesize that a single-session would produce an increase in EEG-based metrics by altering the functional brain state, we would argue that over the course of training, the brain would respond differently due to learning and the amount of energy needed to accomplish the same task would be reduced, or distributed.

## 2 Methods and materials

**Fig. 1:**
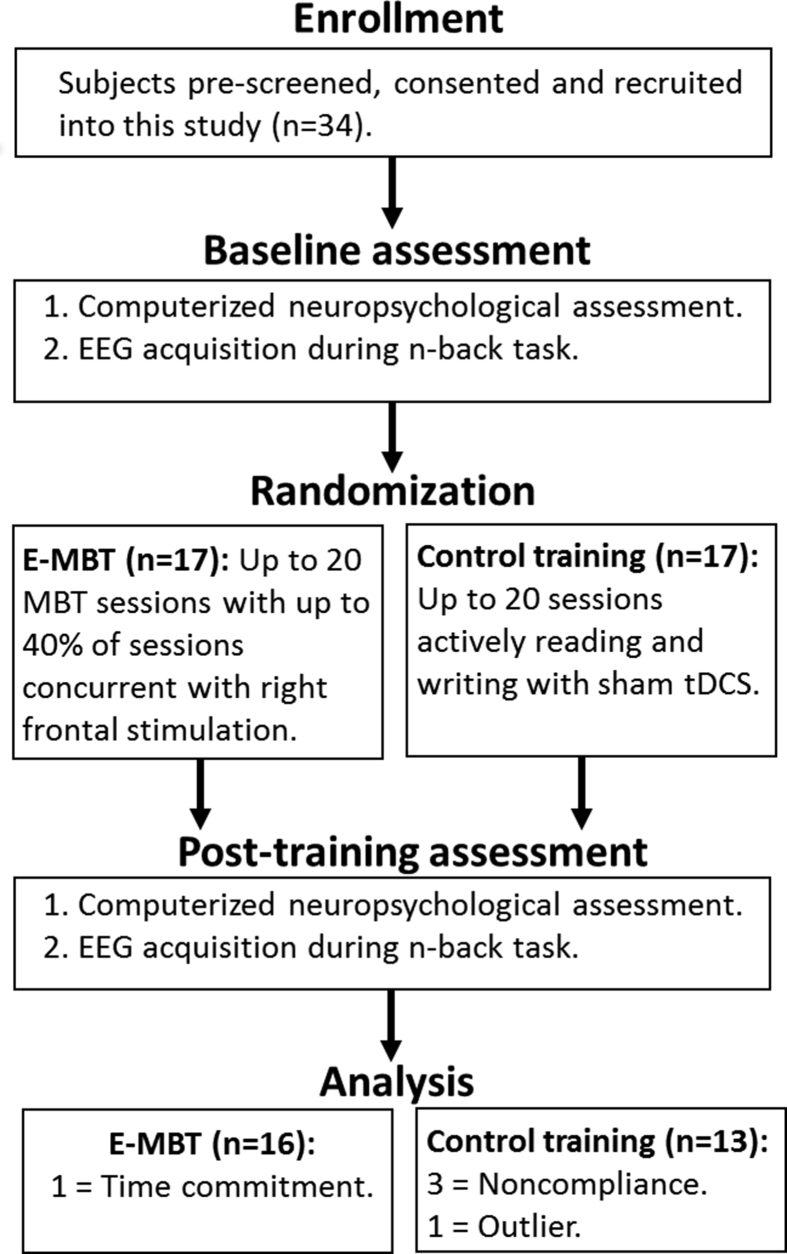
Flow Diagram of Study Methodology. Analysis boxes at bottom show number of subjects excluded for each group and reason for exclusion.

A total of 34 healthy adults were sampled from a larger multi-site study, Multifaceted Intervention for Robust, Adaptive Reasoning and Problem-solving (ARP)-Focused Customized Learning and Enhancement, or MIRACLE, which broadly assessed intervention-related effects on fluid intelligence. For summary purposes, Fig. 1 displays a flow chart of study-related activities. All participants gave written and oral consent to participate in this study, which was approved by Chesapeake Institutional Review Board (IRB): 2014-131270006-2 and by our main campus IRB at the University of New Mexico (UNM). Subjects were randomly assigned to either MBT with active tDCS (2.0 mA) or a control training condition with sham tDCS (0.1 mA). The current study only sampled subjects recruited from the UNM site due to the availability of EEG equipment. Groups were matched for age, gender and Shipley-2 IQ (intelligence test). Participants were recruited from the larger Albuquerque area. Eligibility criteria included: age of 18–50, right-handed, have received a degree from a four-year college or were enrolled in a four-year degree program with at least two years of coursework and English as a native language. Exclusionary criteria included: currently on psychotropic medication, history of neurological or psychiatric conditions, more than 1 hour per week of cognitive training (including MBT) over the last month and/or prior brain stimulation. Four participants were excluded from the study (due to noncompliance or inability to meet the time commitment). Three subjects from the Control group were dropped from the study after the second training session due to noncompliance issues (i.e., all three subjects missed more than 2 scheduled appointments and did not respond to e-mails or phone calls) and one subject from the eMBT group left the study before starting treatment due to competing time commitments. Of the subjects who completed treatment, one outlier who was omitted from subsequent analyses (in the Control group) that positively skewed the shape of the distribution on several variables (both Shapiro-Wilk and Kolmogorov-Smirnov tests were significant, *p* < 0.01 and was 3 SD lower than the group mean on each variable, likely indicating a lack of effort), leaving a total of 29 subjects for subsequent analysis.

### 2.1 Experimental procedures

Participants completed computer-based assessments (programmed in E-prime) before and after training. Between 0 and 2 days after the baseline/post-training neuropsychological assessments, EEG data was acquired while participants performed 1-, 2- and 3-back tasks. Cognitive training (either eMBT or Control) began immediately following the baseline EEG session and continued 5 days per week, for 4 weeks of training. Post-training EEG assessment was then completed 1–4 days after the final training day. All Research Assistants were blind to participant group designation.

### 2.2 Transfer tasks

Tests were randomized and counterbalanced for the pre-training (baseline) assessment, but the order was preserved at the post-training assessment for each subject. The computer-based assessment included O-span (Unsworth et al., 2005), S-Span (Unsworth et al., 2005), Raven's progressive matrices (RPM) (Raven, 2003) and Shipley-2 IQ (Shipley et al., 2009). The O-span required participants to remember the correct sequence of letters (ranging between 3 and 7 items), which were interleaved with trials that asked participants about the veracity of a math equation (i.e., true or false) (Unsworth et al., 2005). After each math trial, subjects were asked to recall, in order, the preceding letters. Scores were calculated by summing the number of letters correctly recalled in the correct order. In a similar fashion, but within the spatial domain, the S-span required participants to memorize and reproduce the locations of the highlighted boxes that appear in a 4 × 4 grid, which were interleaved with trials that asked participants to determine if designs were symmetrical along a vertical axis. Scores were calculated by summing the number of spatial locations correctly recalled in the correct order. The RPM task required participants to identify the missing item in a complex pattern. This task was designed as a measure of abstract reasoning (Raven, 2003). For each session within the current study (i.e., baseline and post-training), 18 unique patterns were randomly presented as a 3 × 3 square matrix with items presented in black on a white background along with 8 possible answer selections (Raven, 2003). For Shipley-2, the vocabulary and abstract reasoning form were used.

### 2.3 Transcranial electrical mindfulness-based training: eMBT

Participants logged onto a website developed and maintained by Charles River Analytics (CRA), Inc, which provided guided mindfulness meditation sessions. Participants were instructed to listen to guided mindfulness meditation recordings for 30 minutes per day, 5 days per week, for 4 weeks. The recording consisted of a guided meditation from experienced mindfulness training practitioners at the Massachusetts Center for Mindfulness. There were two types of MBTs, FA-type and OM-type, available to participants at each training session. Providing a choice of meditation type was desirable to account for individual-differences in meditation preference and to mirror real world settings where individuals would select from a range of possible practice types. It was further expected that allowing for choice and variety in meditation type would increase retention. Due to technical issues with the hosting website, data for which type of meditation session (FA or OM) was chosen by each participant were not collected and therefore data were collapsed across each training-type chosen. While there is evidence that both OM and FA are distinct techniques and both meditation types have been shown to produce performance differences compared to control groups, the difference between each method on WM performance has yet to be fully investigated. Given that the two types of meditation techniques have different effects on cognitive processes, the type of meditation technique a subject chooses (and the number of times one chooses one technique over another) is one possible confound of this study. However, it is worth noting here that there have been several studies that reported negligible effects between meditation types for novice mediators on certain metrics. For instance, Valentine and Sweet (1999) compared OM and FA meditators on a sustained attention task and found that the two groups did not differ in performance, but only when the stimulus was expected. In a more recent study, FA and OM were compared on a modified-version of the attention network task (ANT). While both types of meditation improved conflict resolution compared to a relaxation control group, there was no difference between the two meditation types (Ainsworth et al., 2013). Nonetheless, the current results should be interpreted in light of this potential confound of combining meditation techniques.

In addition, a fraction of participants (25%) also attended a weekly voluntary 50-minute mindfulness webinar which allowed participants to ask questions and provide feedback about their practice (N.B. there was no significant differences in demographics or performance measures between these subjects and others who did not attend the webinars). The mindfulness webinars were led by experienced mindfulness practitioners.

**Fig. 2:**
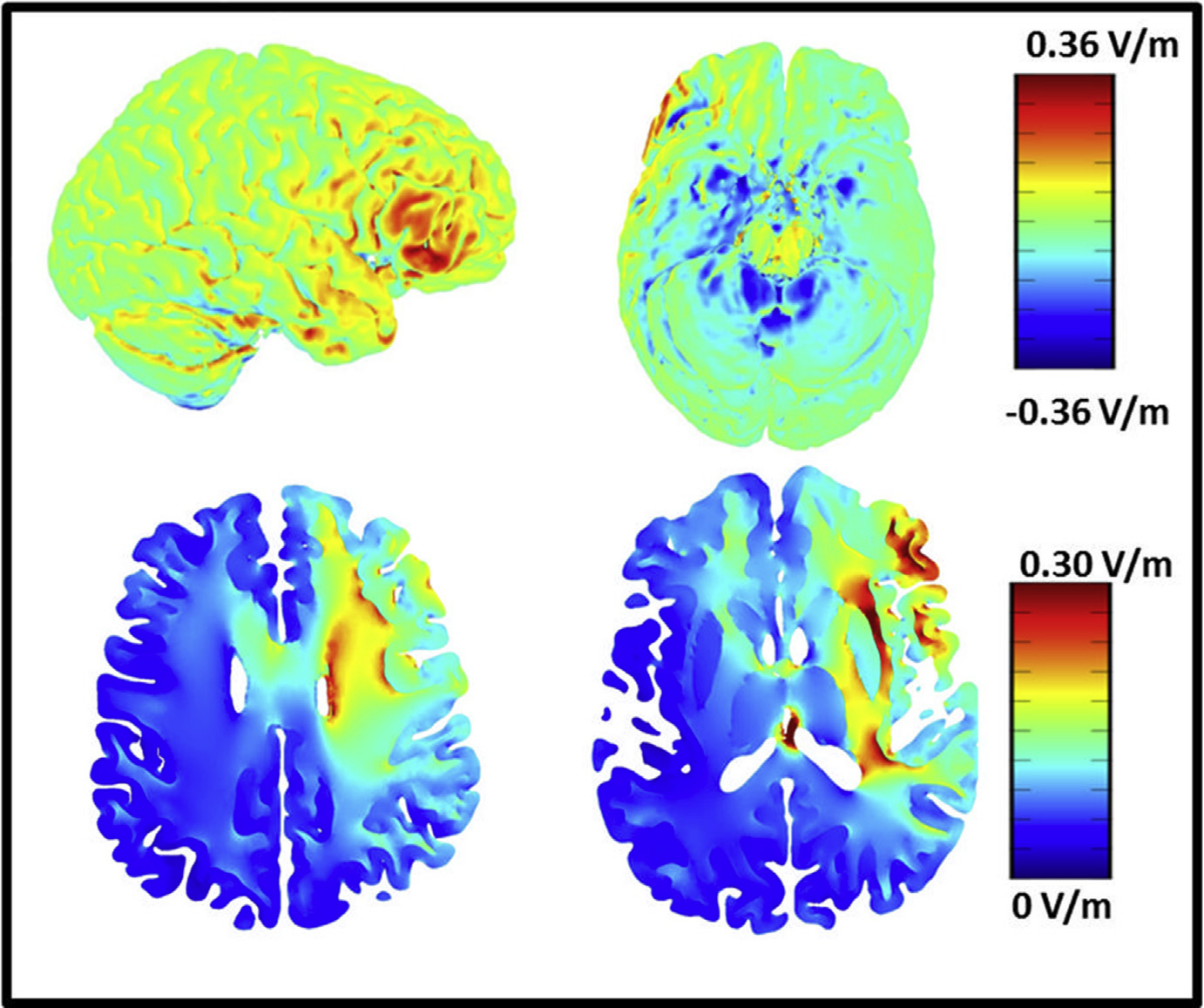
Modeled current distribution of right frontal tDCS montage used during MBT. Modeled current distribution of F10 (anode) and contralateral upper bicep (cathode) placement (Bikson et al., 2012; courtesy of Alexander David and Marom Bikson, City College New York).

Two of the five weekly MBT training sessions took place in the laboratory with concurrent tDCS. The other three took place at home or another quiet space. Because the right IFG plays a critical role in attentional control (Petersen and Posner, 2012; Hugdahl et al., 2015; Hampshire et al., 2009, 2010; Clark et al., 2012; Coffman et al., 2012; Yang et al., 2016), we placed the anode electrode over F10 (or rIFG) and the cathode electrode over contralateral upper-arm. With this placement, we intended to preferentially modulate excitatory signaling in the right-lateralized attention network. Furthermore, by removing the direct influence of the cathode on cortical activity, this montage allows for a more precise evaluation of anodal tDCS on rIFG cortical excitability. Fig. 2 displays the current distribution of the active tDCS montage (Bikson et al., 2012), which highlights the excitability of the right IFG, right insula and other regions thought to be involved in mindfulness practice (Malinowski, 2013; Lazar et al., 2005).

Of these in-lab tDCS sessions, the eMBT group received 2.0 mA of tDCS for 30 minutes. We selected a current strength of 2.0 mA for this protocol as it has been found to me more effective than lower current strengths to modulate attention and learning in previous studies (Clark et al., 2012). Furthermore, given the observed enhanced effect of 2 mA tDCS on oscillatory activity and working memory, compared to 1.0 mA (Hoy et al., 2013) and in previous studies showing evidence for a facilitation of learning and improved attention (Coffman et al., 2012), we used 2 mA current strength in our active condition. Note that if participants were not being able to arrive and/or reschedule within a week, these sessions were omitted altogether for that participant. The goal was to ensure and control for the total amount of time the participants were enrolled in the study and engaged in training.

Furthermore, the tDCS sessions were spaced out this way (in contrast to having all MBT session concurrent with tDCS) both for safety and to control for any possible tDCS-related re-bound effects. For safety, Palm et al. (2008) found that in all subjects undergoing 2 mA reported extensive redness and intracutaneous changes similar to the shape of the electrodes on the 4th consecutive day. The authors concluded that tDCS-induced adverse events may be more likely to occur while stimulating at higher intensities for a longer period of time. Furthermore, another reason for spacing the tDCS sessions was to ensure we did not introduce any confounding effects that are produced by the interacting effects of tDCS on meta-plasticity (see Abraham, 2008 for review).

### 2.4 Control training with sham tDCS

An “active” control task was used to control for test-retest (practice-related) effects on outcome measures and to ensure control subjects were engaged in the experiment and interacted with study personnel for the same amount of time (and potential confluence) as the eMBT group (Shipstead et al., 2012). Participants assigned to the Control group were also told that the control task may improve cognitive skills to ensure similar expectations about training benefits as the eMBT group. These participants logged onto the CRA website that provided various images and passages of text. Participants were asked to view the pictures/passages and write text passages about them for 30 minutes per day, 5 days per week, for 4 weeks of training. Like the eMBT group, of the five control training sessions per week, two were also completed in the lab; however, the Control group received 0.1 mA of stimulation for 30 minutes, which has been shown to be an effective sham protocol in that it induces physical sensation without altering cortical excitability or behavior (Hampshire et al., 2009, 2010).

It is important to note here that there are other “active” control activities that could be used here to compare with MBT, such as, writing in a diary and relaxation training, but there remains further research to assess a properly matched control group for meditation. Furthermore, it is possible that current activity of our control group is not free of any null effects on cognition and may also have an effect on WM performance. Accordingly, any differences observed between groups on the outcome measures of interest would be indicative of the difference within the treatment-related components of the groups themselves and these potential confounds (practice, expectations of a benefit, placebo and the Hawthorne effect) could be reduced.

For both groups, a tracking log of when and for how long participants logged in was kept and reviewed to assess compliance. Table 1 displays the average duration of training within each group. Although, for the at-home sessions, there were no bio-metrics, such as, eye-tracking, tomographic motion detector, accelerometer, video camera, or other objective signals, to determine whether the participants followed instructions as requested, participants were required to press a button at the end of each session indicating that they were present and completed the training. The amount of time it took for participants to press the button was logged and monitored to identify whether participants were not on task. Due to multiple resource limitations, the results of this study are limited to the combined effects of MBT and tDCS compared to baseline metrics and compared to post-training measurements from this active control with sham tDCS group.

**Table 1:** Mean (SD) and corresponding statistics on sample demographic information.

	Control n = 13	eMBT n = 16	Comparisons^a^
Age (years)	26.6 (4.2)	28.4 (6.7)	*t = 0.75, p = 0.45*
College Education (years)	5.2 (1.5)	6.1 (1.8)	*t = 1.24, p = 0.21*
Estimated IQ	111.2 (6.6)	112.0 (8.8)	*t = 0.26, p = 0.79*
Sex (M|F)	8|5	10|6	*χ*^*2*^*= 0.54, p = 0.45*
Number of training sessions	15.8 (2.8)	16.2 (3.8)	*t = 0.33, p = 0.74*
Number of tDCS sessions	7.2 (1.2)	7.1 (0.8)	*t = 0.10, p = 0.91*
Attrition	3	1	*χ*^*2*^*= 1.13, p = 0.60*

a Two-sample t-tests and chi-square tests were computed to test any differences across each of the groups displayed. No statistically significant differences observed.

### 2.5 tDCS materials

An ActivaDose II Iontophoresis Delivery Unit (Activatek, Salt Lake City, UT) was used to deliver current for this study. A double blinding procedure was accomplished by a coded switch box used in our prior research (details described here Coffman et al., 2012). In short, participants were randomly assigned a stimulation code (a number between 1 and 6), which designated whether active or sham stimulation was received. Both the participant and the research assistant were blinded to the link between the assigned number and the stimulation condition itself.

For tDCS preparation, 11 cm^2^ Amrex A102 square sponge electrodes were moistened with saline and placed near the participant's right sphenoid bone corresponding to 10–20 EEG location F10 (anode) and the left lateral upper bicep muscle (cathode). All participants were asked to report physical sensations (itching, tingling and heat/burning) at the site of electrodes on a 11-point Likert scale. The sensation questionnaire was designed to collect information regarding the participant's sensation during and after the application of tDCS. In particular, participants were asked to describe their physical sensations at approximately 1, 5 and 30 minutes after the start of tDCS to monitor participant ratings for itching, tingling and heat/burning on a 11-point Likert scale. However, in order to avoid interrupting the MBT session, sensation data were acquired only at the first and last time points for the eMBT group. A repeated measures ANOVA was used to assess group differences across the 8 sessions, with sensation type (3 levels) and within-session time points (2 levels) as the within-subjects factor and group as the between-subject factor.

There was only 1 participant who reported a 7 or higher in one of the sessions (i.e., endorsed significant discomfort and wanted the session to stop). This training session was continued without stimulation as implemented in our previous study using the same sensation questionnaire and sham stimulation protocol (Coffman et al., 2012).

#### 2.5.1 N-back task during EEG

In the n-back task, participants were presented with a sequence of letters (500 ms duration, 1400 ms ISI, 1.5° visual angle) and asked to press a button with the right thumb if the current letter matched the one presented *n* letters prior in the sequence. The first 10 letters of the alphabet were selected as the possible stimuli. For this study, there were three experimental blocks that progressed in difficulty, starting with 1-back, then 2-back and finally 3-back. This ordering of experimental blocks was the same for all subjects at baseline and post-training. For each block, 35 targets and 70 non-targets were presented (total of 105 stimuli). Participants used their right thumb to respond to the target trials only. Responses faster than 100 ms were discarded. The task was administered using Presentation 16.5 (Neurobehavioral System Inc., Berkeley, C.A.) and behavioral dependent variables (accuracy and RT) were extracted from log files using Excel VBA programming.

As post-hoc exploratory analyses, signal detection statistics were also extracted. For the signal detection measure, the value *d'* was calculated as the difference between the signal (targets) and noise (non-target) distributions (Excel function: NORMSINV (Target) – NORMSINV (Non-target)). The value for response bias (*β*) was calculated as -d'*0.5*(NORMSINV (Target) + (NORMSINV (Non-target))). Standard adjustments were made when accuracies were 1 or when false alarms were 0.

#### 2.5.2 EEG acquisition and preprocessing

EEG data were acquired using a BioSemi 128-channel ActiveTwo system using a restricted 10-10 montage. Bipolar electro-oculogram (EOG) recordings were also acquired, with electrodes placed below the left eye and at the right outer canthus. Electrocardiogram (ECG) recordings were also obtained. All signals were digitized at 1,024 Hz with 24-bit AD conversion. Subject-specific EEG electrode locations were obtained using the Polhemus FastTrak system.

EEG data were pre-processed using the MATLAB toolboxes EEGLAB (http://sccn.ucsd.edu/eeglab/) and ERPLAB (http://erpinfo.org/erplab). Data from each channel were high-pass filtered at 0.01 Hz and DC offset was removed to eliminate DC voltage offset and other slow drifts in the data. Channel locations acquired at EEG preparation were then applied to each participant's dataset. Noisy channels were visually detected, removed from each dataset and then interpolated by surrounding electrodes and Independent Component Analysis (ICA) was used to remove signal artifacts caused by ocular and cardiac signals. Preprocessed continuous EEG data were then referenced to the averaged reference of all scalp electrodes. Epochs were created around the target stimuli (−200 to 1400 ms) and data were baseline corrected to the pre-stimulus interval. Missed response trials and trials containing signals greater than ±80μV were rejected. All single trial epochs were then low-pass filtered at 30 Hz prior to averaging. P3 amplitudes were calculated as the average voltages from 300–650 ms at electrode sites Pz, Fz and F10. While this P3 time window was fixed across all subjects, this interval captured the peak for each subject (ranged from 312–548 ms), as conducted in a previous study (Saliasi et al., 2013). Pz and Fz were selected for analysis to evaluate canonical EEG locations for frontal and parietal P3 amplitude and theta power during WM and F10 was selected to evaluate any possible localized tDCS-related effects.

Time–frequency measures were obtained by convolving stimulus-locked single-trial data from all electrodes with complex Morlet wavelets. Frequency ranged from 1 to 30 Hz in 30 logarithmically spaced steps, with a constant 4 cycle wavelet, which resulted in estimates of instantaneous power (the square of the complex convolution signal, which was then averaged across trials). Averaged theta-based activity was then extracted between 3.8 and 7.5 Hz (log spaced) at the same time window as P3 amplitude extraction (300–650 ms), which was fixed across all subjects. Baseline correction to the time-frequency power was implemented using the recommended methods described by Grandchamp and Delorme (2011) as this method has been shown to outperform traditional baseline correction methods (using pre-stimulus interval). More specifically, power values at each time-frequency point were normalized to the entire trial interval (−200 to 1400 ms) at each trial, which converted power to decibel (dB) scale to further account for power-law scaling of oscillations in different frequency bands. All time-frequency analyses were conducted using recommended MATLAB functions described and published by Cohen (2014), which were appropriately modified for this study.

### 2.6 Statistical comparisons

All statistical comparisons were evaluated in this study using repeated measures ANOVAs, as implemented in SPSSv22 (IBM SPSS Inc., Armonk, NY). The assumptions of normality, homogeneity of variances and equality of the covariance matrices were evaluated using the Shapiro-Wilk and Kolmogorov-Smirnov tests, Levene's test of equality and Box's test, respectively. After the removal of 1 outlier (from the Control group), all tests to evaluate the assumptions of the factorial ANOVAs were non-significant (*p*'s > 0.01). Repeated measures ANOVA was as an appropriate model for several reasons. For example, the upper and lower 95% confidence levels did not include a value of 100% nor negative values (which would otherwise bias the mean estimate, are not possible values of accuracy), especially for the 2- and 3-back conditions. Furthermore, as described above, the assumption of homogeneity of variance and covariance was not violated.

Two-sample t-tests and χ^2^ tests were computed to assess group differences on the baseline demographic and personality trait measures. To test within-subject comparisons (baseline vs post-training and WM load) and between-subject comparisons (Control vs eMBT) on both n-back performance and EEG data, 2 × 2 × 3 repeated measures ANOVAs were computed, with group assignment as the between-subjects factor and time and WM load (1-back, 2-back and 3-back) as within-subject factors.

### 2.7 Exploratory analysis of task transfer

To explore whether the training-related gains observed on the n-back task were related to general gains in WM (S-span and O-span) and fluid IQ (RPM and Shipley IQ), relative change scores ((post-training − baseline)/baseline) were computed for each n-back dependent variable that showed a significant difference between groups in the previous analyses and each of the proposed transfer tasks. Pearson correlations were computed to test if there were positive associations, such that an increase in a relative change score in one variable would correspond to an increase in a relative change score in the other transfer task. Similarly, correlations were assessed between the number of eMBT sessions and these relative change scores to examine any possible training-related dose effects.

Lastly, given our hypothesis regarding training-induced effects on performance and on the corresponding EEG responses, a priori pair-wise t-tests were conducted even without the presence of a statistically significant interaction within the omnibus test, which were Bonferroni corrected (α ≤ 0.01 or 0.05/3 since there were 3 pair-wise comparisons at each WM load).

## 3 Results

**Fig. 3:**
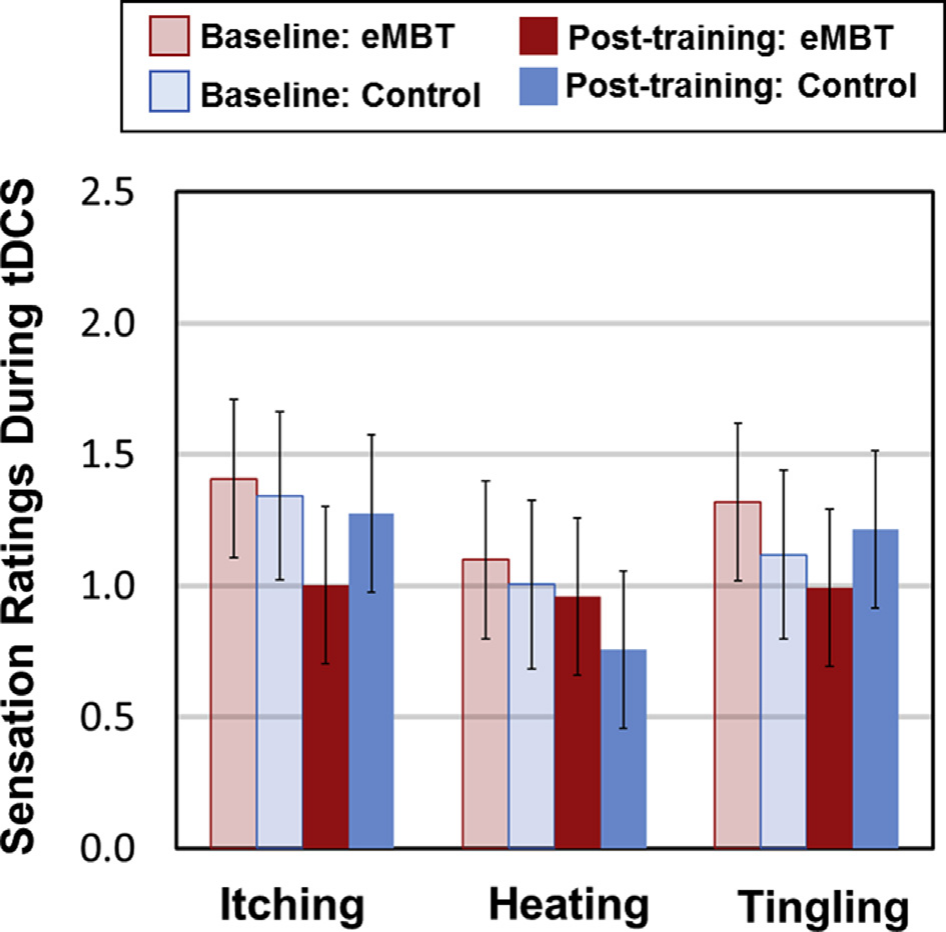
Mean, SE and statistical comparisons on tDCS sensations collapsed across sessions. Mean sensation ratings were averaged across tDCS sessions at ∼1 minute after tDCS was “turned on” (labeled ‘Baseline’) and again after the tDCS session, which was ∼30 mins (labeled ‘Post-training’) for each group.

Sensation rating results are presented in Fig. 3. There were no statistically significant differences between groups on any of the sensation ratings (*p's* > 0.10).

In short, there were no statistically significant differences between eMBT and Control groups on any of the sensation ratings (*p*'*s* > 0.10) collapsed across the 8 tDCS sessions.

Table 1 displays all demographic information for all subjects who were included in subsequent analyses. Two-sample t-tests and χ^2^ tests showed that there were no statistically significant differences between groups on any of the baseline demographic and personality trait measures (all *p'*s > 0.19).

### 3.1 N-back performance

**Fig. 4:**
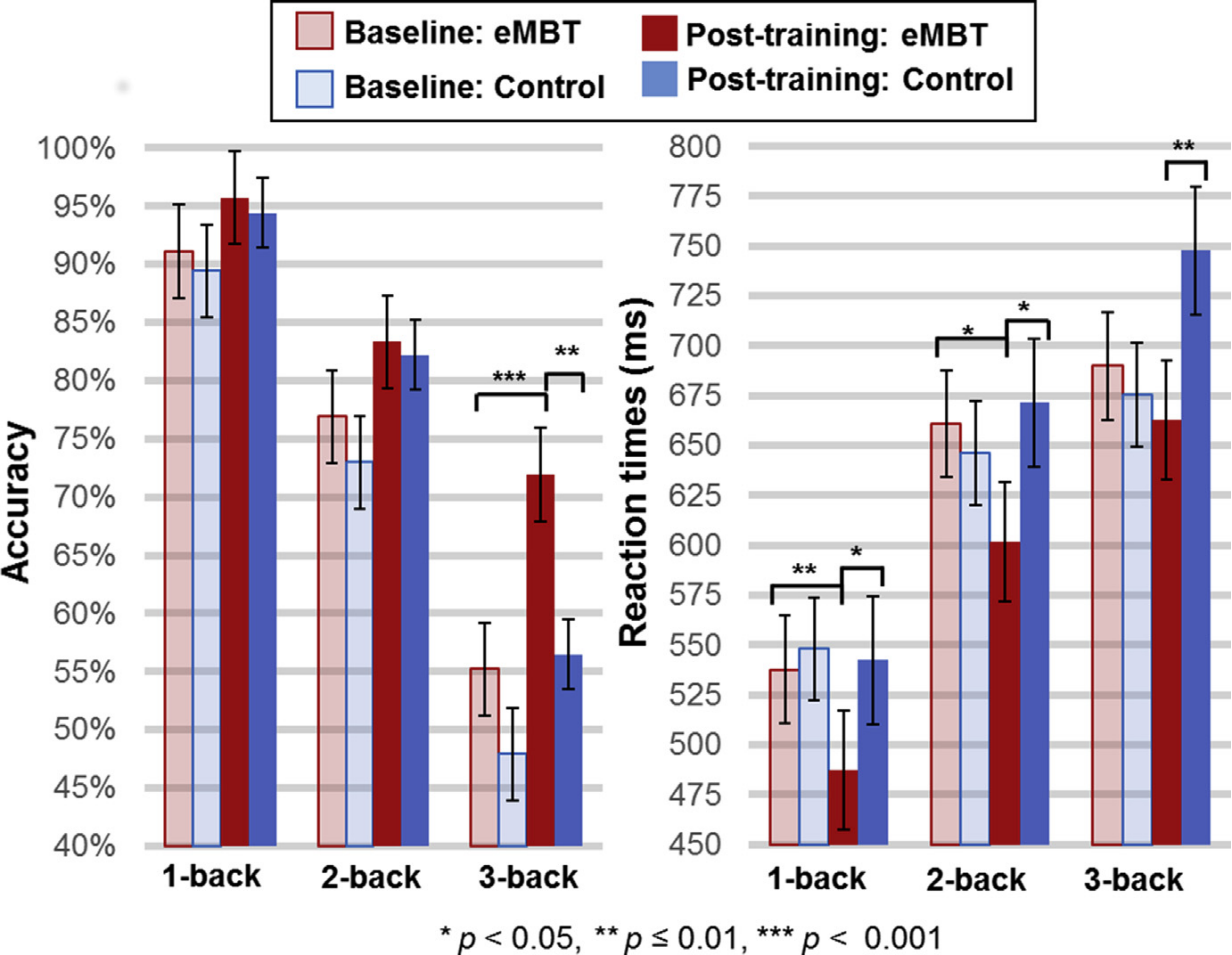
Mean, SE and statistical comparisons of working memory performance within each group at baseline and post-training. Baseline data are represented by transparant fill and post-training data by solid fill both for the eMBT (red) and Control (blue) groups.

The group means and standard errors (SE) for each of the WM load conditions are displayed in Fig. 4. For the accuracy measures (i.e., hit rate), there was a main effect of time (post-training > baseline, *F*(1, 28) = 28.36, *p* < 0.001, *η*_*p*_^*2*^ = 0.54) and WM load (*F*(2, 27) = 169.67, *p* < 0.001, *η*_*p*_^*2*^ = 0.88). Collapsed across groups, pairwise comparisons between conditions showed that 1-back accuracy was greater than 2-back accuracy (*t*(28) = 16.63, *p <* 0.001) and 2-back accuracy was greater than 3-back accuracy (*t*(28) = 13.07, *p* < 0.001). These results are consistent with practice-related effects and the increased difficulty of this task as a function of each WM load condition. Although there was not a statistically significant group × time × load interaction (*p* = 0.10, *η*_*p*_^*2*^ = 0.14), this effect was marginal. Given that the sample size is relatively small in this study and therefore, maybe underpowered and in order to evaluate the primary hypothesis, pair-wise t-tests were conducted, which showed a significant increase on post-training 3-back performance in the eMBT group compared to the Control group (*t*(28) = 2.42, *p* < 0.01), but not for the 1- or 2-back conditions (*p*'s > 0.49). Furthermore, post-training 3-back accuracy was significantly increased relative to baseline within the eMBT group (*t*(28) = 4.07, *p* < 0.001). There were no differences between groups on baseline accuracy scores for any of the WM load conditions (*p'*s > 0.26), nor between baseline and post-training accuracy within the Control group for any of the WM load conditions (*p*'s > 0.10).

For the n-back RT measures, there was a main effect of WM load (*F*(2, 27) = 60.15, *p* < 0.001, *η*_*p*_^*2*^ = 0.72). 1-back RT was faster than 2-back RT (*t*(28) = 9.73, *p* < 0.001) and 2-back RT was faster than 3-back RT (*t*(28) = 7.75, *p* < 0.001). There was also a group × time interaction (*F*(1, 28) = 9.27, *p* = 0.006, *η*_*p*_^*2*^ = 0.28), where follow-up one-way ANOVAs showed that post-training RTs in the eMBT group were faster than the Control group RTs when collapsing across WM load conditions (*F*(1, 29) = 13.94, *p* < 0.001, *η*_*p*_^*2*^ = 0.22). Furthermore, collapsed across WM load conditions, within-subject comparisons showed that the eMBT group responded quicker after training relative to baseline (*F*(1, 15) = 6.37, *p* = 0.01, *η*_*p*_^*2*^ = 0.25). It is worth noting that this difference was most pronounced for the 1-back (*t*(15) = 3.36, *p* = 0.002), with a difference in the same direction for the 2-back (*t*(15) = 2.38, *p* = 0.03) condition. There were no differences in baseline RTs between groups (*p* = 0.85, *η*_*p*_^*2*^ = 0.01), nor between time points within the Control group (*p* = 0.12, *η*_*p*_^*2*^ = 0.09).

### 3.2 Post hoc exploratory analysis on signal detection measures

**Fig. 5:**
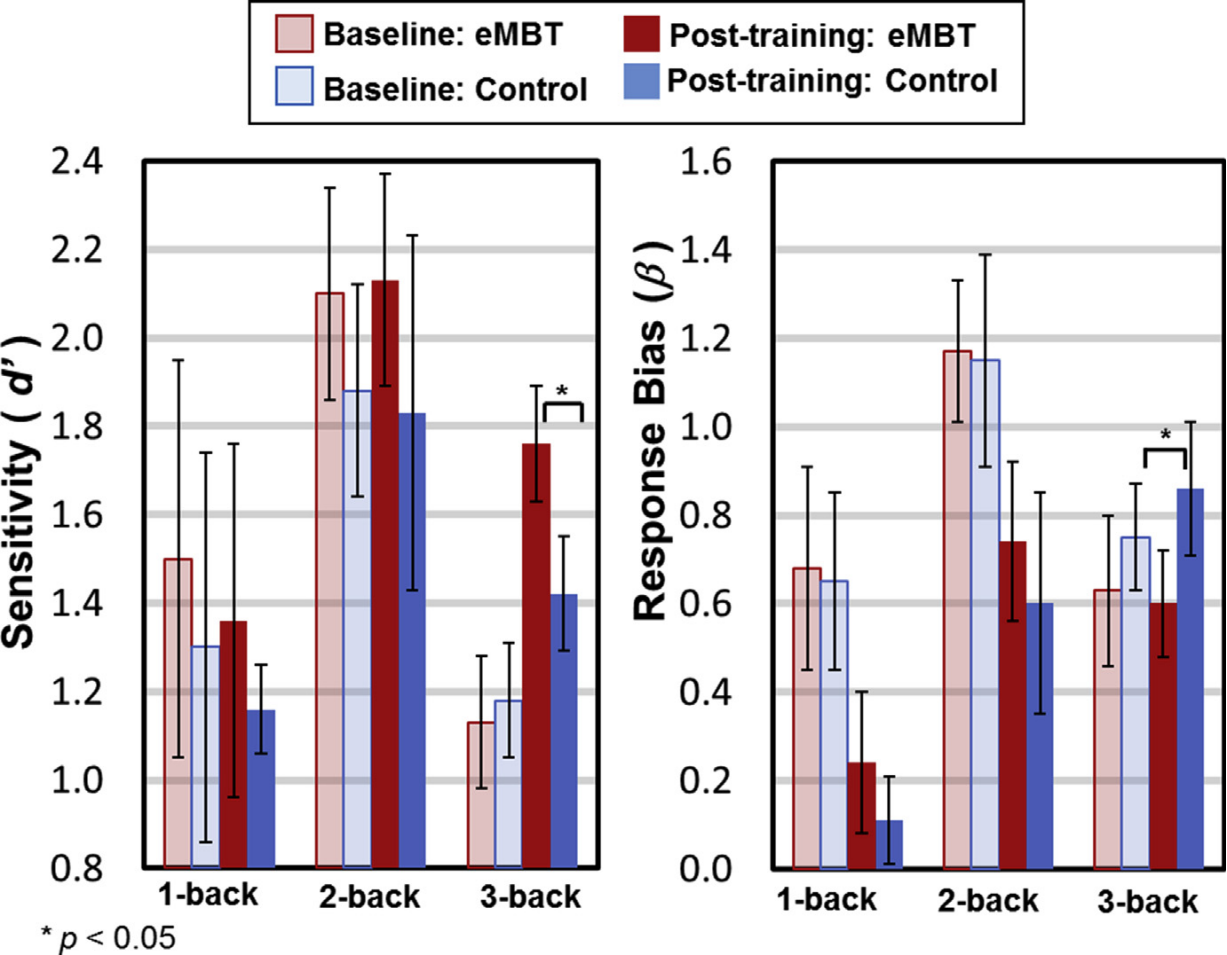
Mean, SE and statistical comparisons of signal detection measures of working memory performance within each group at baseline and post-training. Baseline data are represented by transparant fill and post-training data by solid fill both for the eMBT (red) and Control (blue) groups. For *d′* scores, the higher values indicate a better ability to distinguish and detect target and non-target stimuli and thus an increase in signal discrimination. For response bias (*β*), a higher score indicates a more “cautious” response tendency (i.e., avoiding commission errors). A lower score indicates a more “free” response tendency to ensure a response is made to most or all targets.

Group means and standard errors (SE) for the sensitivity (*d'*) and response bias (*β*) indices for each of the WM load conditions are displayed in Fig. 5. For *d'*, a main effect of WM load (F(2, 27) = 58.79, *p* < 0.001, η_p_^2^ = 0.39). Collapsed across groups, pairwise comparisons between conditions showed that the 2-back was higher than 1-back (*t*(28) = 10.16, *p* < 0.001) and the 2-back conditions (*t*(28) = 8.26, *p* < 0.001). To specifically test whether there were any group differences, post hoc test were conducted and found a statistically significant increase in *d'* during the 3-back condition for the eMBT group compared to the Control group, *t*(28) = 2.12, *p* < 0.05. Furthermore, the post-training *d'* scores during the 3-back condition in the eMBT group increased relative to baseline, *t*(28) = 3.98, *p* < 0.001.

For the n-back response bias (*β*), there was a main effect of WM load (*F*(2, 27) = 13.06, *p* < 0.001, *η*_*p*_^*2*^ = 0.31). 2-back *β* was higher than 1-back (*t*(28) = 5.73, *p* < 0.001) and marginally higher than 3-back (*t*(28) = 1.75, *p* = 0.06). To specifically test whether there were any group differences, post hoc test were conducted and found a statistically significant increase in *β* during the 3-back condition for the Control group relative to the eMBT group, *t*(28) = 2.06, *p* < 0.05.

### 3.3 P3 amplitude

**Fig. 6:**
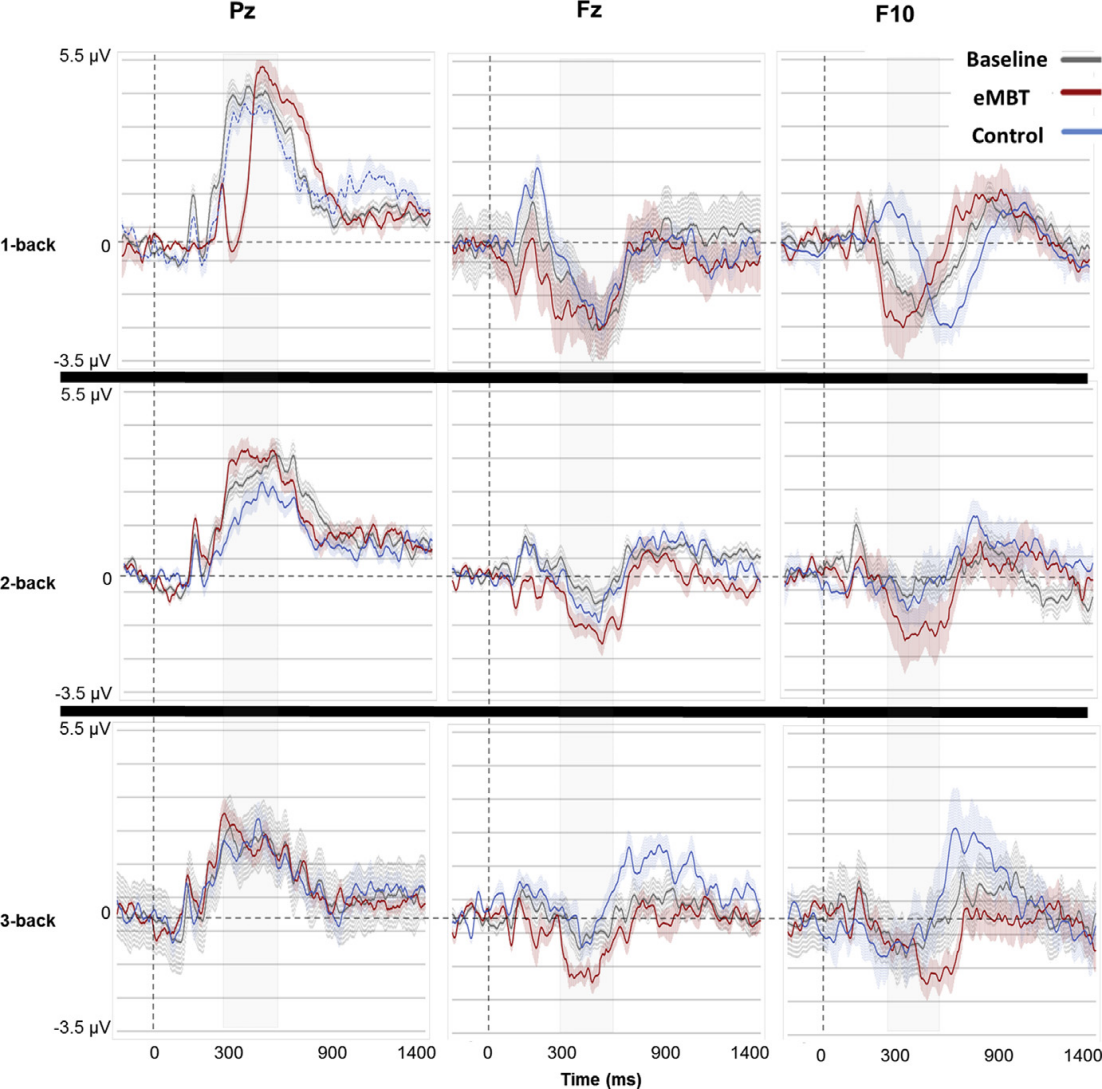
Baseline and group-specific post-training ERP waveforms for Pz, Fz and F10 as a function of working memory load. P3 activity was averaged between 300–650 ms (displayed as gray transparent time window).

**Fig. 7:**
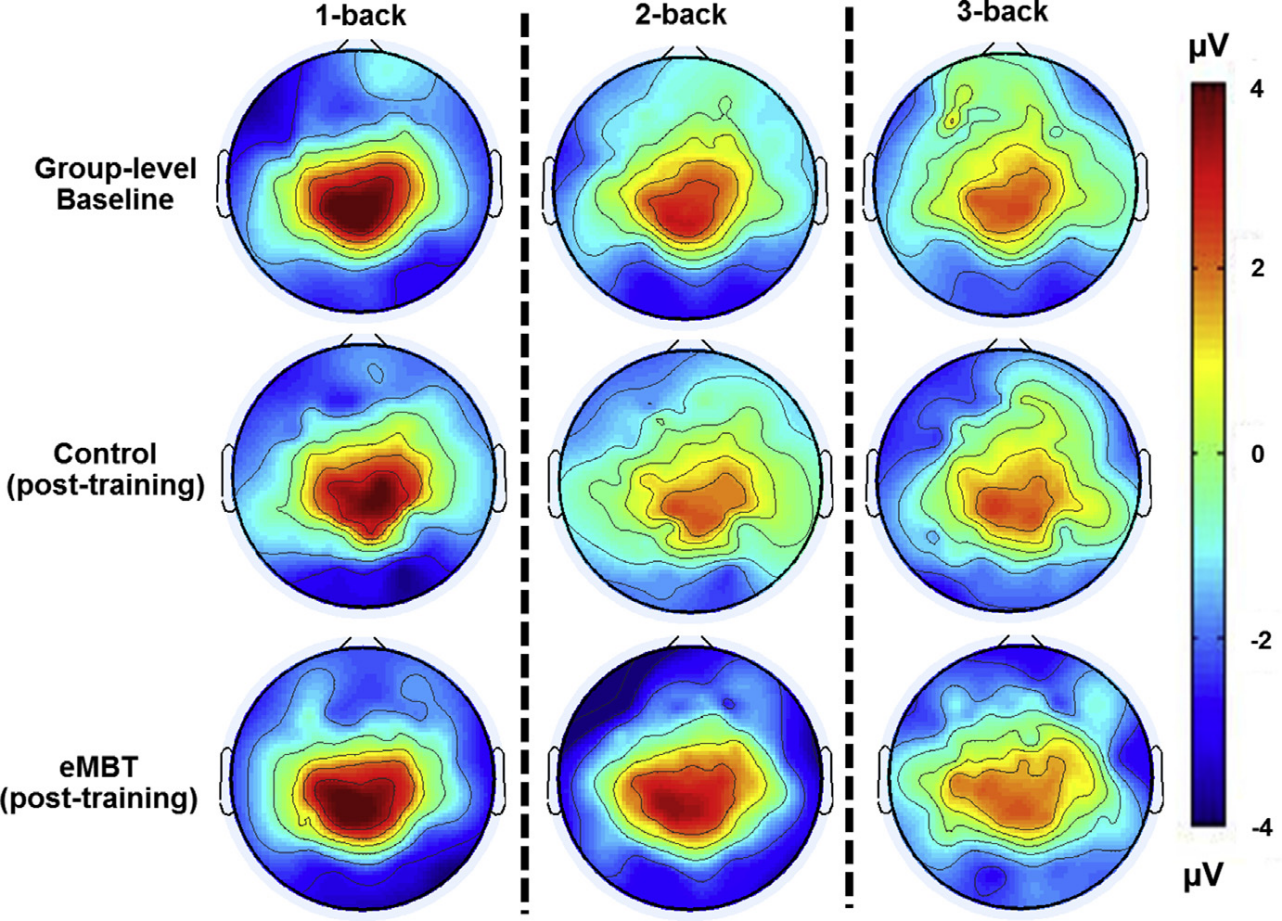
Baseline and group-specific post-training P3 topology as a function of working memory load. P3 amplitude topology averaged between 300–650 ms and decreased as a function of WM load.

**Fig. 8:**
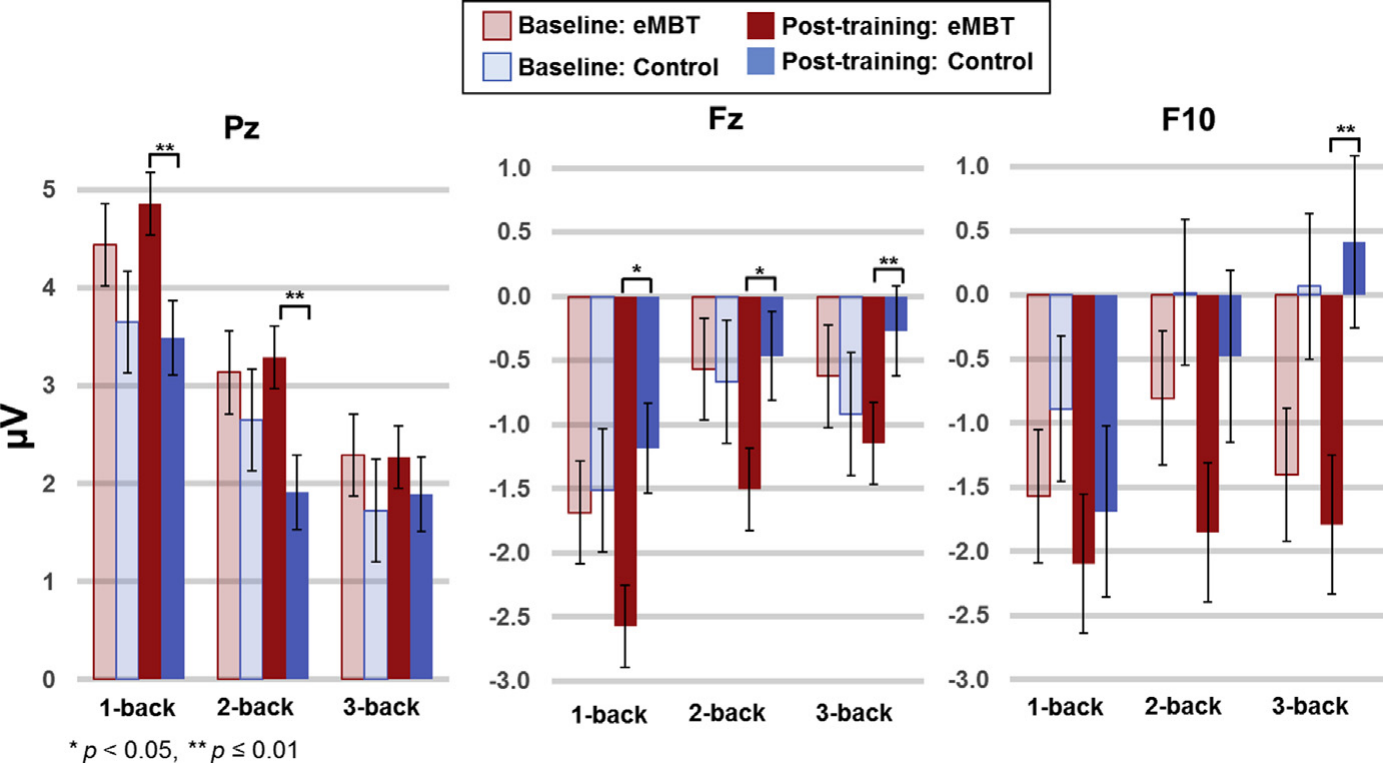
Mean, SE and statistical comparisons of P3 amplitude for all working memory load conditions and electrode sites of interest within each group at baseline and post-training. Baseline data are represented by transparant fill and post-training data by solid fill for both the eMBT (red) and Control (blue) groups. P3 activity was computed as the average between 300–650 ms.

For each subject, there was an average of 2.3 trials rejected (*SD* = 1.1) due to artifacts, which did not differ between groups or between baseline and post-training sessions (*p* > 0.67). The baseline and group-specific post-training P3 waveforms and topology are displayed in Figs. 6 and 7, respectively. Results show the expected P3 topology, with activity centralized over posterior electrodes, with activity more dispersed to frontal sites (e.g., Fz and F10) as a function of WM load. Group means, SEs and statistical comparisons for each of the WM load conditions at each electrode site of interest are displayed in Fig. 8.

At electrode Pz, there was a main effect of WM load on P3 amplitude (*F*(2, 27) = 118.18, *p* < 0.001, η_p_^2^ = 0.84). Collapsed across groups, pairwise comparisons between conditions showed that 1-back P3 amplitude was greater than 2-back amplitude (*t*(28) = 6.89, *p* < 0.001) and 2-back amplitude was greater than 3-back amplitude (*t*(28) = 4.82, *p* < 0.001), which was similar to the WM performance accuracy results shown above. Pair-wise t-tests were conducted to evaluate the hypothesized differences within and between groups. There was a significant increase in post-training 1-back responses (*t*(27) = 3.07, *p* = 0.005) and 2-back responses (*t*(27) = 2.57, *p* = 0.01) in the eMBT group compared to the Control group. There were no differences between groups on baseline Pz responses in any of the WM load conditions (*p*'s > 0.10), nor between baseline and post-training Pz responses within the Control group in any of the WM load conditions (*p*'s > 0.27).

At location Fz, there was also main effect of WM load, such that P3 approached a positive mean amplitude as a function of increased WM load (*F*(2, 27) = 12.17, *p* = 0.002, η_p_^2^ = 0.34). Collapsed across groups, pairwise comparisons between conditions showed that 1-back P3 amplitude was greater than 2-back amplitude (*t*(28) = 3.65, *p* = 0.001) but 2-back amplitude was not significantly different than 3-back amplitude (*t*(28) = 0.13, *p* = 0.63). There was also a group × time interaction, *F*(2, 27) = 4.41, *p* = 0.04, η_p_^2^ = 0.16. Follow-up one-way ANOVAs showed that post-training P3 amplitude in the eMBT group was more negative compared to the Control group when collapsing across WM load conditions (*F*(1, 28) = 10.54, *p* = 0.003, η_p_^2^ = 0.32). It is worth noting that these differences were most pronounced for the 3-back condition (*t*(27) = 3.06, *p* = 0.006). Furthermore, collapsed across WM load conditions, the eMBT P3 responses at Fz were attenuated after training relative to baseline (*F*(15) = 5.75, *p* = 0.03, η_p_^2^ = 0.18). There were no differences in baseline Fz responses between groups (*t*(27) = 1.21, *p* = 0.88), nor between time points within the Control group (*p*'s > 0.38).

Finally, at location F10, there was a main effect of WM load, similar to Fz in that the positive amplitude increased as a function of increased WM load, (*F*(2, 27) = 6.24, *p* = 0.02, η_p_^2^ = 0.21). Collapsed across groups, pairwise comparisons between conditions showed that 1-back P3 amplitude was greater than 2-back amplitude (*t*(28) = 2.33, *p* = 0.03), but 2-back amplitude was not significantly different than 3-back amplitude (*t*(28) = 0.27, 0.78). Although there were no significant interactions, pair-wise t-tests were conducted to assess our proposed hypothesis, which showed a significant attenuation on post-training 3-back responses (*t*(*27*) = 2.89, *p* = 0.008) in the eMBT group compared to the Control group. There were no differences between groups at baseline for any of the WM load conditions (*p*'s > 0.24), nor between baseline and post-training accuracy within the Control group on any of the WM load conditions (*p'*s > 0.21).

### 3.4 P3 range theta power

**Fig. 9:**
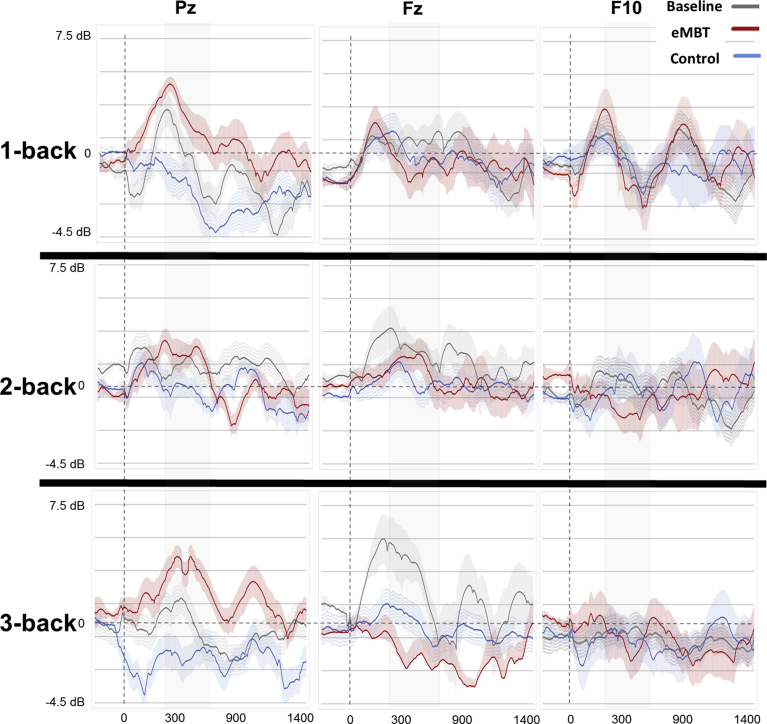
Baseline and group-specific time-frequency theta power for each working memory load condition at each electrode site of interest. Theta power was decibel scaled and baseline corrected from −200 to 1400 ms (per trial, prior to averaging). For all subsequent comparisons, theta power was averaged between 300–650 ms (displayed as gray transparent time window) and from 3.8–7.5 Hz (log spaced).

**Fig. 10:**
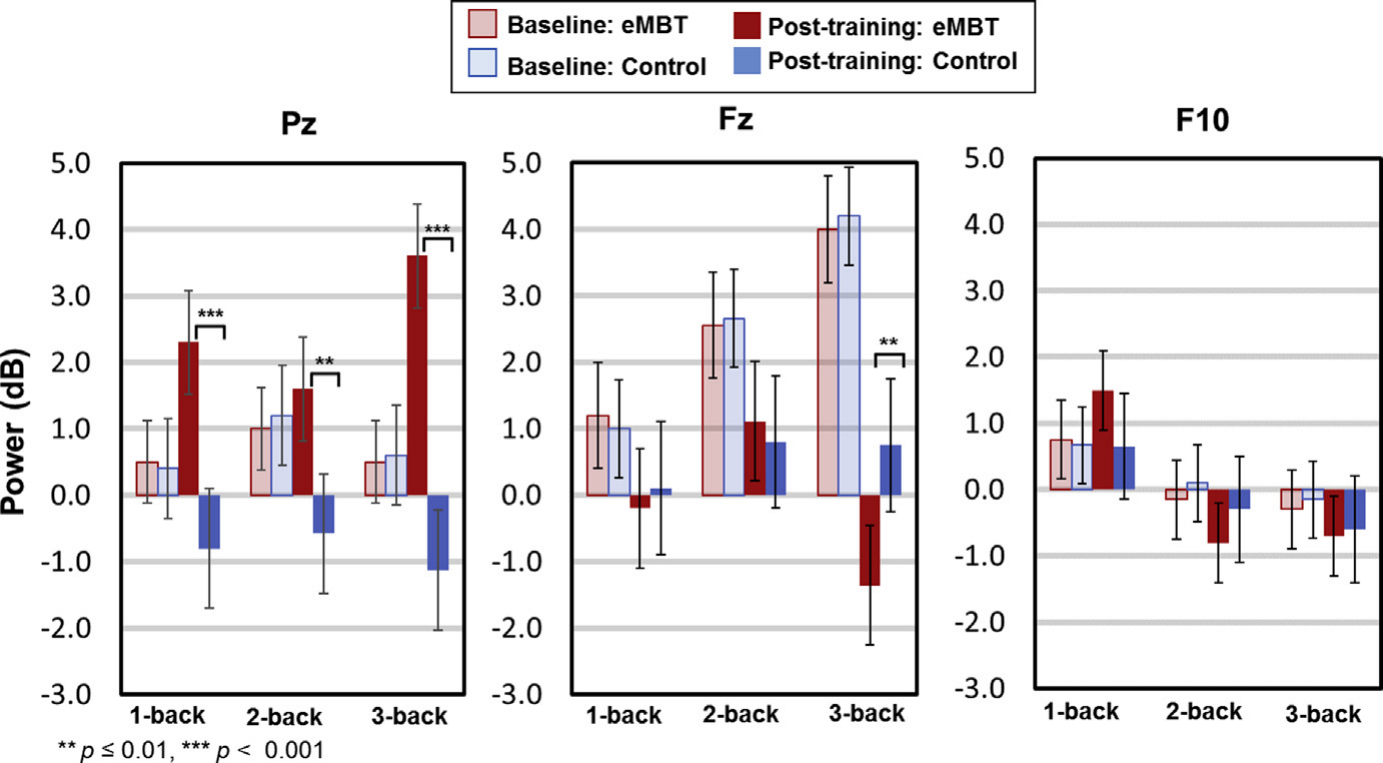
Mean, SE and statistical comparisons of theta power for all working memory load conditions and electrode sites of interest within each group at baseline and post-training. Baseline data are represented by transparant fill and post-training data by solid fill for both the eMBT (red) and Control (blue) groups. Mean theta power was averaged between 300–650 ms and from 3.8–7.5 Hz (log spaced).

The baseline and post-training theta time power time courses for each WM load condition are displayed in Fig. 9. Results show a consistent pattern of increased power as a function of WM load during the baseline, specifically for Fz and Pz electrode sites. Theta power group means, SEs and statistical comparisons for each of the WM load conditions and each electrode site of interest are displayed in Fig. 10.

For location Pz, there was a group × time interaction (*F*(1, 28) = 36.15, *p* < 0.001, η_p_^2^ = 0.62). A follow-up one-way ANOVAs showed that post-training power in the eMBT group was increased compared to the Control group when collapsing across WM load conditions (*F*(1, 28) = 13.21, *p* < 0.001, η_p_^2^ = 0.39). It is worth noting that these differences were most pronounced for the 3-back condition (*t*(27) = 3.62, *p* < 0.001). Furthermore, collapsed across WM load conditions, Pz theta power in the eMBT group was significantly increased after training relative to baseline (*F*(1, 28) = 29.62*, p* < 0.001, η_p_^2^ = 0.58). Although the following effect is non-significant after Bonferroni correction, it is important to note that for the 2-back condition only, there was an unexpected baseline difference during the pre-stimulus interval between groups (*t*(27) = 2.35, *p* = 0.03). However, there were no differences within the P3 range across groups (*p*'s > 0.86).

At electrode site Fz, there was a group × time × WM load interaction (*F*(2, 27) = 5.60, *p* = 0.03, η_p_^2^ = 0.21). Although there were no statistically significant 2-way interactions (*p's* > 0.26), follow-up pair-wise t-tests showed that post-training theta power in the eMBT group was decreased in the 3-back condition only compared to baseline (*t*(15) = 3.89, *p* = 0.001), but only marginally significant compared to post-training theta power in the Control group (*t*(27) = 2.06, *p* = 0.05). There were no differences in baseline Fz power between groups (*p*'s > 0.08), nor between time points within the Control group (*p*'s > 0.28).

For power at electrode site F10, there were no main effects, interactions or pair-wise difference between groups nor between time points (*p*'s > 0.38).

### 3.5 Exploratory task transfer effects

**Fig. 11:**
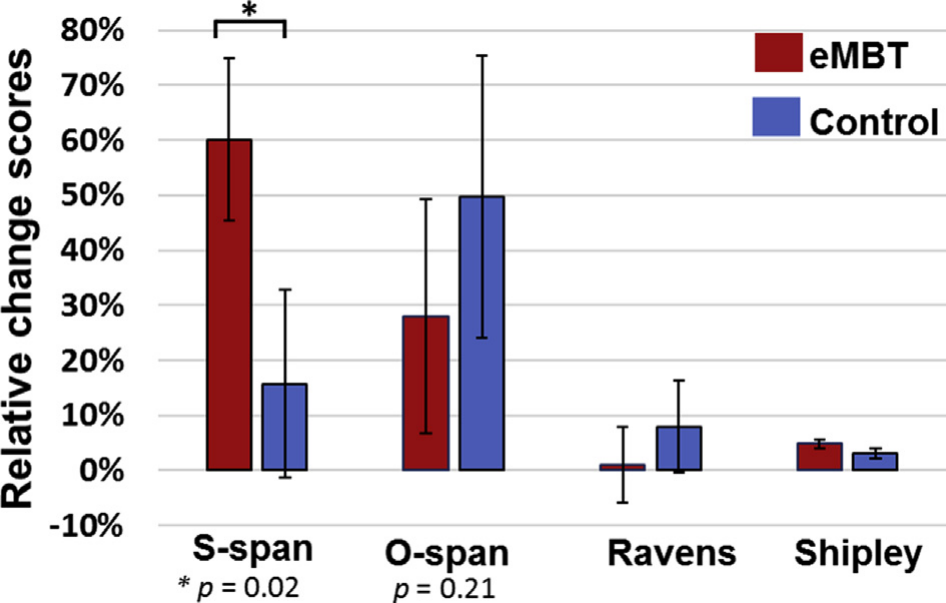
Mean, SE and statistical comparisons of near and far transfer relative change scores for each group. Relative change scores were computed as the difference between post-training and baseline scores divided by the baseline scores, which were then converted to percentage points.

Group means and SE of each transfer task and personality traits of interest are displayed in Fig. 11. Between-subject comparisons showed a marginally significant increase in S-span scores in the eMBT group compared to the Control group (*t*(27) = 2.16, *p* = 0.02). Note that this effect did not survive multiple-comparisons correction (Bonferroni corrected *p* < 0.0125, or 0.05/4 since there were 4 transfer tasks). There were no other statistically significant differences observed between groups in the other transfer measures (*p*'*s* > 0.21).

For the performance data, there was a significant association between the relative change in 3-back accuracy scores and S-span accuracy scores collapsed across both groups, (*r*(29) = 0.49, *p* = 0.009). However, this correlation was primarily driven by the eMBT group (*r*(16) = 0.58, *p* = 0.02) as there was no association observed within the Control group, (*r*(13) = 0.07, *p* = 0.84). There were also no statistically significant correlations observed between the n-back and O-span, nor between the RPM and IQ relative change scores collapsed across groups, nor within either group (*p'*s > 0.66). To test whether the zero-order correlations observed in the eMBT group were statistically higher compared to the control group, the observed zero-order correlation was transformed into Fishers' Z-scores and a one-tailed z-test was calculated between groups as follows:

$${\text{Z}}_{\text{observed}}=\frac{{\text{z}}_{\mathrm{group}1}–{\text{z}}_{\mathrm{group}2}}{\sqrt{\frac{1}{N1-3}+\frac{1}{N2-3}}\;}$$

Where z_group_ corresponds to the Fisher's Z transformed correlation for group 1 and group 2 and N is the number of subjects within each group.

The correlation magnitude (S-span x 3-back change scores) in the eMBT was only marginally higher compared to the control group, Z = 1.42, *p* = 0.078.

For the ERP data, there was an eMBT group-specific relationship between S-span relative change scores and relative changes in P3 amplitude during 3-back performance in the F10 electrode site (*r*(16) = −0.55, *p* = 0.04). There were no other correlations observed between the EEG measures and the transfer relative change scores.

**Fig. 12:**
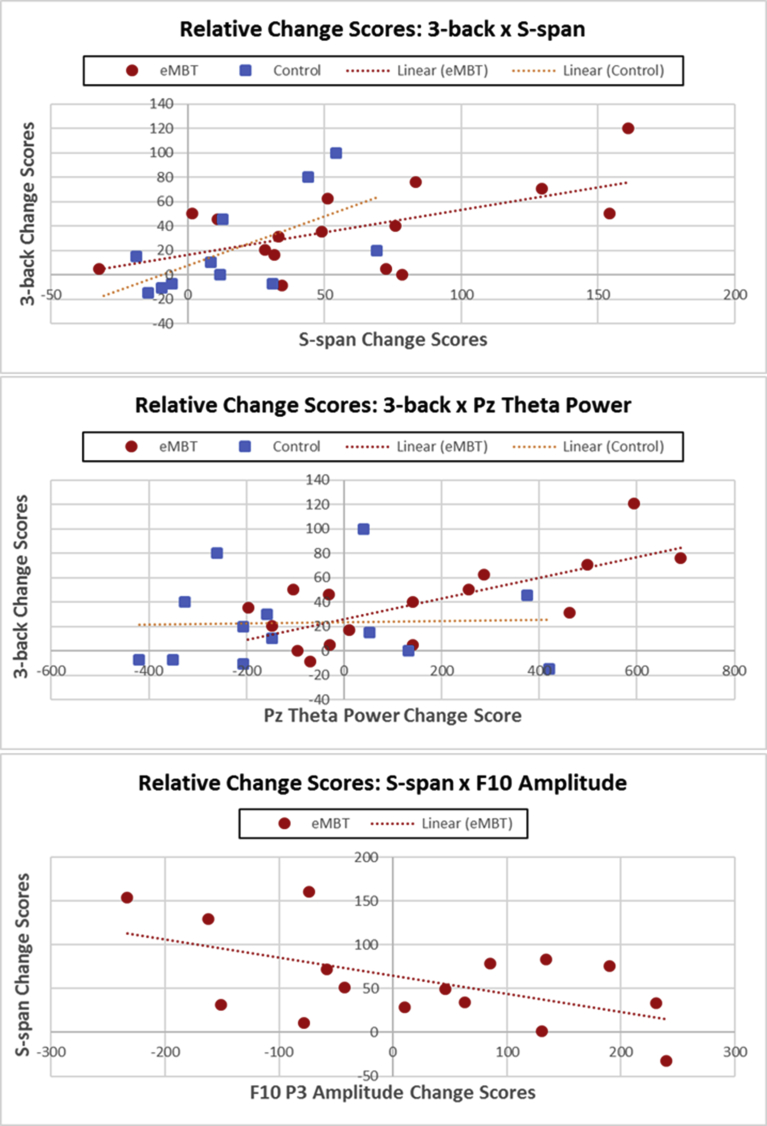
Scatterplots for each statistically significant bivariate correlation among relative change scores. The eMBT group is displayed as red circles and the control group is shown as blue squares. The top scatterplot shows the relation between 3-back accuracy and S-span change scores for eMBT and for controls. The middle scatterplot displays the relation between 3-back accuracy and Pz theta power (during 3-back condition) for eMBT and for controls. The bottom scatterplot shows the relation between S-span and F10 P3 amplitude (during 3-back condition) change scores for the eMBT group only.

The only variable that correlated with relative change scores (for accuracy) in the 3-back condition was theta power at the Pz electrode site, collapsed across groups (*r*(29) = 0.45, *p* = 0.02). Again, this correlation was primarily driven by the eMBT group (*r*(16) = 0.71, *p* = 0.006), as there was no association observed within the Control group (*r*(13) = 0.37, *p* = 0.86). The correlation magnitude (3-back x Pz theta power change scores) in the eMBT was higher compared to the control group, *Z* = 1.87, *p* = 0.03. All statistically significant bivariate correlations are displayed in Fig. 12.

## 4 Discussion

The present study recruited subjects from a larger multi-site study to specifically examine the combined effect of mindfulness-based training (MBT) combined with tDCS (eMBT) on behavioral and neurophysiological indices of WM and attentional allocation. While it was beyond the scope of the present study to test the independent contribution of each component of eMBT (e.g., the effects of tDCS on MBT alone), the current study was designed to specifically evaluate our hypothesis-driven training parameters, comprising the potential compatibility of MBT with tDCS across time (4-weeks) and compare these effects between an active control with sham tDCS group.

It is also important to note that the current study allowed participants to choose between the focused attention-type (FA) and open monitoring-type (OM) meditation technique, which accommodated individual-differences in meditation preference and was used to increase retention. Overall, this study is an important contribution to the scientific community as it demonstrates the technique's multi-session feasibility, establishes effect sizes on objective measures of attentional allocation in a nonclinical population and revealed valuable information that can help guide future, related studies.

### 4.1 Enhancements of working memory load performance

Although there was not a significant group by time interaction within the omnibus test, pairwise comparisons revealed effects consistent with our first hypothesis, namely that the eMBT group demonstrated training-related improvements in WM capacity. This was evinced by increased 3-back accuracy relative to baseline performance and compared to post-training 3-back performance in the Control group. Given that the observed increases in hit rate were largest at the most demanding WM load condition, the observed training-related increase in accuracy may reflect an alteration in the range of capacity limits in the eMBT group.

However, there was not a statistically significant increase in the eMBT group for the intermediate conditions of the n-back task (i.e., the 1- and 2-back). A previous study reported a significant increase in extended hit rate during an adaptive 2-back task (Zeidan et al., 2010) and suggested that the MBT group maintained focus under conditions that required rapid stimulus processing. The present findings are consistent with this indication of enhanced rapid stimulus processing, but that this effect was localized to the most challenging condition of the task procedures in the current study.

This enhancement in WM capacity was further evinced by faster response times across WM load conditions after training in the eMBT group compared to the Control group, with the most robust effects observed also in the more difficult 2- and 3-back WM load conditions. However, for the 3-back condition, it is important to note that this RT effect size is influenced by slower post-training RTs in the Control group. This effect was not present for the intermediate n-back conditions, however. Further, the eMBT group did respond quicker across all (three) n-back conditions relative to baseline performance. Together, these results suggest that the eMBT group improved in overall WM capacity and attentional states (Oken et al., 2006). Interestingly, in the current study, increased accuracy during the 3-back condition was observed without the predicted tradeoff for slower RTs (Wood and Jennings, 1976). Although speculative, it is possible that the combined effect of eMBT may have altered the threshold for simultaneously performing more accurately without losing efficiency in processing speed (Rioult-Pedotti et al., 1998).

Consistent with these results we also observed an increase in post-training d' scores during the 3-back condition in the eMBT group compared to the Control group. This finding suggests an enhanced perceptual sensitivity in the eMBT, specifically during the 3-back condition. Interestingly, there was no change in response style for the eMBT group, but there was an increase in response bias (β) in the Control group. An increase in response bias in the Control group indicates response style consistent with a “cautious” (or lowered) expectation toward target probability. This increase in response bias in the Control group, but not the eMBT group, may have implications for eMBT decreasing fatigue and/or perceived effort in the face of increased workload. Altogether, the present results suggest an increase in the WM capacity constraints and state-dependent information processing in the eMBT group. Further research is needed to examine whether the speed-accuracy tradeoff is influenced by various types of tDCS protocols and/or different types of MBT techniques.

### 4.2 Facilitation of neuronal efficiency as measured with P3 amplitude

In the present study, alterations in the magnitude of the P3 ERP component was interpreted as an attention-related measure of neuroplastic change. Consistent with previous studies, P3 amplitude to targets in the current study decreased as a function of WM load, indicating more efficient neural processing resulting from greater resource allocation during more demanding WM load conditions (Kok, 2001; Polich and Criado, 2006; Gevins and Cutillo, 1993; Cabeza et al., 2002; Gaspar et al., 2011).

After training, the eMBT group displayed an increase in posterior P3b amplitude for the less demanding WM load conditions compared to the Control group. Consistent with our hypothesis, the eMBT group also exhibited a simultaneous attenuation of P3a amplitude across all WM load conditions at the frontal electrode sites (i.e., Fz and F10), particularly for the most demanding WM load condition (i.e., 3-back). These results suggest a differential P3 response pattern in the distribution of EEG topology, where a heightened posterior response was recruited during low-demand WM load conditions and an attenuated P3 frontal response was found during high WM load conditions.

Previous studies have reported a similar reversal in P3 topology and magnitude and suggested that it may be due to the functional distinction between these two regions at varying levels of WM demands (Gevins and Cutillo, 1993; Vogel et al., 2005; Lakey et al., 2011). That is, an intensified posterior P3b response during low-demand WM load conditions is thought to reflect an EEG topology associated with high performing individuals who achieve better performance by paying more attention to basic stimulus matching, which requires less resource allocation (Vogel et al., 2005). Thus, the current findings suggest that eMBT may further improve information processing by enhancing the ability to concentrate or focus during tasks that require minimal resources.

However, during high cognitive load conditions, a neural system may recruit a more widely distributed network with reciprocal connections between frontal and parietal association areas (Kok, 2001; Polich and Criado, 2006). Given the changing patterns of brain activation at varying levels of cognitive load (Gevins and Cutillo, 1993; Fehr, 2013; Rypma and D'Esposito, 1999; Callicott et al., 2003; Karlsgodt et al., 2009), the neural efficiency hypothesis postulates that a higher cognitive capacity level is associated with more efficient brain functioning (see Neubauer and Fink, 2009 for review). Individuals who score high on difficult tasks (e.g., WM tasks) tend to rely less on the frontal cortex as they gain mastery over a cognitive or complex procedural skill, whereas the inefficient use of frontal circuits — observed as high levels of activity during less demanding tasks — is associated with worse performance. Consistent with this hypothesis, one study found that older adults performed worse during WM performance and exhibited increased midline frontal P3 amplitude compared to younger adults (Saliasi et al., 2013). The authors suggested that the increased P3 amplitude reflected inefficient use of neural resources.

Thus, the observed decrease in post-training P3a amplitude at the frontal electrodes in the eMBT group, specifically in the context of high WM load demands, may reflect the activation of a more widespread network involved in attentional capacity and the categorization of task-relevant events (Gevins and Cutillo, 1993). Given the observed frontal decreases in P3a amplitude in the eMBT group, one study found that high capacity performance reflects more efficiency by representing only the relevant items compared to low capacity performance, which is characterized more by inefficient encoding and maintenance of WM on irrelevant items (Vogel et al., 2005). The current findings provide evidence that eMBT may expand WM load capacity constraints by efficiently recruiting multiple brain networks to efficiently allocate attention to relevant information.

### 4.3 Re-distribution of frontal and posterior theta power to further support neuronal efficiency

Consistent with previous studies investigating the relation between WM load and theta power in healthy populations (Sauseng et al., 2005; Onton et al., 2005; Missonnier et al., 2006; Meltzer et al., 2008), time-frequency analysis of the current data revealed increased midline frontal theta power in the P3 range as a function of WM load prior to training. This observation is thought to reflect efficient use of the active maintenance of WM items and continual cognitive engagement (Owen et al., 2005).

After training in the present study, the eMBT group exhibited a decrease in midline frontal theta power during the 3-back condition only, which was inconsistent with our initial hypothesis, where midline frontal theta is typically increased (see Lomas et al., 2015 for review). However, there are a number of methodological differences to consider. Previous studies either examined frontal theta during meditation, during rest (i.e., in the absence of engaging task-related networks), or assessed it immediately (same day) after training. Thus, the present study differs substantially from previous literature since the current group differences in theta power reflects alterations on task-related networks rather than resting-state networks. Furthermore, this task-related activity is thought to be affected by a combined neurocognitive training technique designed to induce long-term neuroplasticity outside the context of state-related MBT changes.

Accordingly, the decrease in midline frontal theta power in the eMBT group was also observed with a simultaneous increase in posterior theta power during the high-demand WM load condition. One possible interpretation is that this pattern of results may reflect an overall enhancement of neural efficiency toward better performance (Neubauer and Fink, 2009). For instance, one study found that MBT follows a non-linear dosage–response curve, such that early skills training may reflect different activation patterns compared to later phases of training and/or more advanced practices (Allen et al., 2012). Thus, it is conceivable that there was an initial increase in frontal theta early in training, which became more distributed throughout the course of training, observed here as a simultaneous decrease in midline frontal theta power and increase in posterior theta power.

Accordingly, there is empirical evidence that emphasizes global efficiency among the functional connectivity between frontal and parietal cortices, which operate more rapidly during more demanding aspects of a WM task (see Deary et al., 2010 for review). A previous study found that parietal activity in the P3 range was related to training-related improvements in processing speed and attentional control, which transferred to performance gains on another attention task (Oelhafen et al., 2013). To this end, the observed recruitment of parietal theta may have afforded better WM performance in the eMBT group. Indeed, relative increases in Pz theta power was correlated with relative gain scores during the 3-back condition. The magnitude of this correlation was statistically larger than the Control group (*p* = 0.03), this result suggests that increased theta power in Pz may be beneficial for engaging (or recruiting) more efficient attention-related processes, which may be influenced by mechanisms that underlie a form of neural adaptation in polysynaptic association cortices within the eMBT group. Future longitudinal studies are needed to track this hypothesized non-linear relationship between the time course of training (including training intensity) and brain energy re-distribution.

### 4.4 Possible training-induced right lateralized bias toward spatial working memory ability

Of the four transfer tasks analyzed in the current study, only S-span performance was significantly increased in the eMBT group compared to the Control group. However, it is important to note that although this effect was statistically significant (relative to baseline), it did not survive multiple comparisons (Bonferroni) correction when considering the other four transfer tasks that were evaluated. Interestingly, S-span relative gain scores were also positively correlated with 3-back relative gain scores in the eMBT group only, suggesting a possible training-induced transfer of WM ability in the eMBT group.

Though speculative, this pattern of results found in the eMBT group with right hemisphere brain stimulation suggests a domain specific training-related improvement in WM ability (Miller and Cohen, 2001; Klingberg, 2010; Shipstead et al., 2012). To this end, it is plausible that the right frontal anodal stimulation applied during MBT in the current study biased an enhancement of right-hemispheric visuospatial WM abilities by strengthening the local neuronal connections supporting a larger, distributed spatial WM network. This is consistent with prior evidence demonstrating that a single session of tDCS is sufficient to change functional connectivity within and between large-scale intrinsic networks (Hunter et al., 2015). Thus, given that right frontal regions are more involved in spatial-location monitoring (Schmiedek et al., 2009) and that tDCS can improve visuospatial WM performance (Jeon and Han, 2012), the observed domain-specific improvement (i.e., increases in S-span, but not O-span performance) in the eMBT group may be attributed to the combined effect of MBT with active right frontal tDCS. Furthermore, this possible lateralized bias towards the right hemisphere may have also affected performance on the O-span task, albeit without a significant reduction in performance. Previous MBT studies have shown a significant increase in this task (Jha et al., 2010; Mrazek et al., 2013). While the Control group showed a trend improvement on the O-span relative to baseline (though not statistically significant), the eMBT group, on average, showed almost no effect at on O-span performance relative to baseline. The proposed lateralized bias on visuospatial WM abilities may be influenced by a tDCS-induced modulation on the processes involved in inter-hemispheric functional connectivity (e.g., transcallosal inhibition).

Finally, another finding consistent with this hypothesis was an observed statistically significant association between change scores of S-span performance and change scores in F10 (right frontal) theta power in the eMBT group. This relationship indicates that increases in F10 theta power during the 3-back condition relative to baseline may predict increases in performance on the S-span performance relative to baseline, which may suggest a possible task transfer of working memory in eMBT to the area of brain stimulation.

Altogether, the current findings suggest that the current eMBT protocol may facilitate the implementation of attentional control processing during WM tasks. Future studies are needed to examine whether there are specific effects (as opposed to global benefits across all measures) associated with the amount of MBT and whether domain-specific WM abilities are modulated preferentially by left and right frontal stimulation. Furthermore, studies are also needed to assess dose-related responses (e.g., sham vs. 1.0 mA vs. 2.0 mA tDCS). Other future research is also needed to systematically compare whether the current eMBT is more effective than tDCS and/or MBT alone.

### 4.5 Limitations and recommendations for future studies

There are several important limitations to consider when interpreting the results of this current study. First, the strict inclusion criteria for this study (e.g. education requirements) may limit the generalizability of the current results.

Second, the collection of only two time points to assess neuroplasticity due to training may have reduced power to detect transient or incremental, non-linear effects on neurophysiological measures obtained in this study. Furthermore, it is important to acknowledge that previous studies have demonstrated non-linear effects of tDCS on brain and behavior as a function of exposure (e.g., number of tDCS sessions) and dose response that may have an influence on the current results. For example, previous studies have reported differential effects of current dose and brain pathology (Hoy et al., 2013, 2014), time intervals between multiple tDCS sessions (Monte-Silva et al., 2013), metaplasticity (Carvalho et al., 2015), individual difference in the tonic-phasic balance of neurotransmitters near the stimulating electrode (Nitsche and Paulus, 2000; Plewnia et al., 2013) and the specific cognitive state of the subject before and during brain stimulation (i.e., state dependency hypothesis; Silvanto et al., 2008) and levels of motivation and task difficulty (see Berryhill et al., 2014 for review). While each of these areas of research are essential to better understanding the effects of tDCS on brain and behavior, this research is ongoing and requires a longitudinal and multi-level approach to fully understand the parameter space of these non-linear effects, which will likely play a role in the current results and other future multi-session tDCS studies. An optimal design would include at least 3 to 4 time points to ascertain the incremental changes induced by training.

Third, due to technical issues with the hosting website, data for which type of meditation session (FA or OM) chosen by each participant were not collected and therefore it is impossible to parcel out the variance within eMBT that contributed most to the observed effects.

Fourth, due to multiple resource limitations (e.g., funding, participant availability, data collection time, etc.), the results of this study are limited to the combined effects of MBT and tDCS compared to an active control with sham tDCS conditions. Thus, the lack of data in cells crossing these factors limits our ability to independently assess the additive effects of tDCS on MBT in this study. This would require a number of crossed experimental conditions to fully examine the numerous factors involved, including adding MBT with sham tDCS and Control with active tDCS groups and methods to examine the differential effects of anode and cathode electrodes, along with other relevant variables (e.g., current strength and electrode locations). Nonetheless, the current study provides initial support for a potential compatibility amount MBT with concurrent tDCS to enhance WM and reallocate neuronal resources, but should be considered preliminary until confirmed with a larger sample and more exhaustive study design.

Lastly, due to the lack of statistical power in the current study design (e.g., relatively small N), some of the behavioral and EEG omnibus interactions were not statistically significant, while the hypothesis-driven pair-wise comparisons revealed significant effects. Despite these pitfalls, the current study provided a platform by which to further objectively evaluate the practical utility of this specific neurocognitive training technique, which should be further tested to examine if there are any incremental effects produced by eMBT on performance and brain responses.

### 4.6 Conclusions and future directions

To the authors' knowledge, this is the first published work to objectively examine the working memory (WM) and attentional allocation effects of eMBT compared to an active control group. The current study found an improvement in WM performance using an n-back task and the complex S-span task, suggesting a possible modification to the range of cognitive capacity limits. Additionally, changes in the attention-sensitive P3 component and its theta oscillatory profile revealed a pattern of results consistent with neural efficiency hypothesis within frontal and parietal activity. Furthermore, it was hypothesized that right frontal anodal stimulation may bias an enhancement of lateralized WM ability, observed in the current study as enhanced right-hemispheric visuospatial WM activity. However, due to a limitation in the design of this study, the present results are not able to directly evaluate whether tDCS adds to the already demonstrated benefits of mindfulness training and long-term practice alone. Advancements in this domain of research will ultimately guide future studies with the goal of tailoring neurocognitive training techniques that maximize an individual's cognitive capacity, specifically by expanding his or her skill sets and abilities that can be transferred to various aspects of real-world cognition.

## Declarations

### Author contribution statement

Michael A. Hunter: Conceived and designed the experiments; Performed the experiments; Analyzed and interpreted the data; Wrote the paper.

Gregory Lieberman: Conceived and designed the experiments; Performed the experiments; Analyzed and interpreted the data.

Brian Coffman, Michael C. Trumbo, Mikaela L. Armenta, Charles S.H. Robinson, Anthony J. O'Sickey, Aaron P. Jones: Performed the experiments; Analyzed and interpreted the data.

Matthew A. Bezdek, Victoria Romero, Seth Elkin-Frankston, Sean Gaurino, Leonard Eusebi, Eric H. Schumacher, Katie Witkiewitz, Vincent P. Clark: Conceived and designed the experiments.

### Funding statement

The research is based upon work supported by the Office of the Director of National Intelligence (ODNI), Intelligence Advanced Research Projects Activity (IARPA), via Charles River Analytics (contract #2014-131270006). IARPA paid for the development of intervention, equipment, portions of data collection, and salaries for most people that worked on the project. The views and conclusions contained herein are those of the authors and should not be interpreted as necessarily representing the official policies or endorsements, either expressed or implied, of the ODNI, IARPA, National Science Foundation (NSF), the Army Research Laboratory (ARL) or the U.S. Government. The U.S. Government is authorized to reproduce and distribute reprints for Governmental purposes notwithstanding any copyright annotation thereon. Funding was also provided by the National Science Foundation Graduate Research Fellowship Program (DGE-0903444) and by the National Academies Ford Pre-Doctoral Fellowship to Michael A. Hunter. Part of Gregory Lieberman's funding was sponsored by the Army Research Laboratory (ARL) and was accomplished under Cooperative Agreement Number W911NF-16-2-0149. The funders had no role in study design, data collection, analysis, decision to publish, or preparation of the manuscript.

### Competing interest statement

The authors declare no conflict of interest.

### Additional information

No additional information is available for this paper.
